# Picking pithy plants: Pith selectivity by wild white‐faced capuchin monkeys, *Cebus imitator*

**DOI:** 10.1002/ajp.23549

**Published:** 2023-09-10

**Authors:** Allegra N. DePasquale, Alice C. Poirier, Megan A. Mah, Cinthia Villalobos Suarez, Adrian Guadamuz, Saul Cheves Hernandez, Ronald Lopez Navarro, Jeremy D. Hogan, Jessica M. Rothman, Omer Nevo, Amanda D. Melin

**Affiliations:** ^1^ Department of Anthropology and Archaeology University of Calgary Calgary Alberta Canada; ^2^ Escuela de Ciencias Biológicas Universidad Nacional de Costa Rica Heredia Costa Rica; ^3^ Área de Conservación Guanacaste La Cruz Costa Rica; ^4^ Nature Conservancy of Canada Toronto Ontario Canada; ^5^ Department of Anthropology CUNY Hunter College New York New York USA; ^6^ German Centre for Integrative Biodiversity Research (iDiv) Halle‐Jena‐Leipzig Leipzig Germany; ^7^ Institute of Biodiversity Friedrich Schiller University Jena Jena Germany; ^8^ Department of Medical Genetics University of Calgary Calgary Alberta Canada; ^9^ Alberta Children's Hospital Research Institute University of Calgary Calgary Alberta Canada

**Keywords:** *Cebus imitator*, fallback food, food selection, foraging ecology, zoopharmacognosy

## Abstract

Understanding diet selectivity is a longstanding goal in primate ecology. Deciphering when and why primates consume different resources can provide insights into their nutritional ecology as well as adaptations to food scarcity. Plant pith, the spongy interior of plant stems, is occasionally eaten by primates, but the context is poorly understood. We examine the ecological, mechanical, chemical, and nutritional basis of plant pith selection by a wild, frugivorous‐omnivorous primate (*Cebus imitator*). We test the hypothesis that pith is a fallback food, that is, consumed when fruit is less abundant, and test for differences between plant species from which pith is eaten versus avoided. We collected 3.5 years of capuchin pith consumption data to document dietary species and analyzed “pith patch visits” in relation to fruit availability, visits to fruit patches, and climatic seasonality. We analyzed dietary and non‐dietary species for relative pith quantity, mechanical hardness, odor composition, and macronutrient concentrations. Capuchins ate pith from 11 of  ~300 plant species common in the dry forest, most commonly *Bursera simaruba*. We find that pith consumption is not directly related to fruit availability or fruit foraging but occurs most frequently (84% of patch visits) during the months of seasonal transition. Relative to common non‐dietary species, dietary pith species have relatively higher pith quantity, have softer outer branches and pith, and contain more terpenoids, a class of bioactive compounds notable for their widespread medicinal properties. Our results suggest that greater pith quantity, lower hardness, and a more complex, terpenoid‐rich odor profile contribute to species selectivity; further, as pith is likely to be consistently available throughout the year, the seasonality of pith foraging may point to zoopharmacognosy, as seasonal transitions typically introduce new parasites or pathogens. Our study furthers our understanding of how climatic seasonality impacts primate behavior and sheds new light on food choice by an omnivorous primate.

## Highlights

- We studied plant pith foraging behavior of white‐faced capuchin monkeys to determine if pith is a fallback food, consumed when fruit is less abundant. We unite this with detailed study of pith physical and chemical properties.
- We find that pith is not a fallback food and instead is consumed around seasonal transitions. Dietary piths contain more pith, are softer, and have a more complex, terpenoid‐rich odor profile relative to non‐dietary piths.
- Our results suggest that pith foraging is highly seasonal, selective, and points to a possible role of zoopharmacognosy.

## Graphical abstract

“When and why do white‐faced capuchins eat plant pith? We investigated how ecological variables and plant traits shape patterns of pith consumption. We find that pith foraging occurs primarily during seasonal transitions, and that dietary pith species contain relatively more pith, are softer, and have a different odor profile than non‐dietary species.“

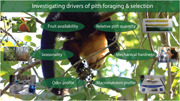

**Abbreviations**
FBF: fallback food
PPV: pith patch visit
PSM: plant secondary metabolites
SSR: Sector Santa Rosa

## 1 INTRODUCTION

How do animals respond when their preferred foods are scarce, yet dietary and nutritional needs must be met? Many primate species prefer ripe fruit when available, yet respond differently to periods of fruit dearth, including changing their ranging patterns (Fiore, 2003), activity budgets (Fan et al., 2013; Stevenson et al., 2000), or diets (Constantino & Wright, 2009; Lambert & Rothman, 2015; Tutin et al., 1997). For example, spider monkeys (genus *Ateles*) and chimpanzees (genus *Pan*) travel long distances or fission into smaller groups to maintain a high‐quality diet, while other primates, such as capuchins (genera *Cebus* and *Sapajus*), tend to maintain fidelity to home ranges and social groups, but shift their diets to include other resources, such as insects and difficult‐to‐access plant parts (Chapman et al., 1995; Fedigan & Jack, 2001; Wrangham et al., 1998). The concept of “fallback foods (FBFs)” has become a popular framework for understanding primate diets (Altmann, 2009). The Fallback Food Hypothesis posits that animals use alternative food sources in inverse proportion to the availability and/or usage of preferred foods to survive periods of food scarcity; these alternative foods tend to be lower quality than preferred foods, either mechanically protected, harder to access, or of lower nutritional quality (Marshall et al., 2009; Marshall & Wrangham, 2007). Because of this critical function, FBFs may disproportionately influence the evolution of behavioral and morphological adaptations; recognizing this role promises to improve our understanding of primate dietary ecology (Lambert & Rothman, 2015; Marshall & Wrangham, 2007; Rosenberger, 2013). It is important to note, however, that the FBF paradigm may not capture the entire range of variation in dietary strategies across primates (Kane et al., 2020; Lambert & Rothman, 2015; McGraw & Daegling, 2012; McGraw et al., 2014). Some primates consume mechanically hard food items habitually as a major component of the diet, rather than as a seasonal fallback food (sooty mangabeys, McGraw et al., 2014), while others willingly leave fruit patches to supplement their diet with non‐fruit items (orangutans, DiGiorgio et al., 2023).

To investigate patterns in the usage and choice of alternative foods, it is useful to understand the timing of their consumption in response to ecological variables as well as the influence of their physical and chemical properties on selectivity (Marshall et al., 2009). For many primates, alternative resources are consumed more when ripe fruit is scarce or unavailable (Lambert & Rothman, 2015; Melin, Young, et al., 2014; Serckx et al., 2015; Wrangham et al., 1991). Other ecological variables relating to climatic seasonality, such as rainfall and temperature, may also influence patterns of resource use by causing heat and/or water stress (Campos & Fedigan, 2009; Chaves et al., 2021) or by shaping resource availability and other ecosystem properties (Mosdossy et al., 2015; Wrangham et al., 1991). Further, the choice of which foods to consume is shaped by the physical and chemical properties of the food, including its relative abundance, mechanical properties, chemical profile (including potential toxins), and nutritional profile (Lambert et al., 2004; Lambert & Rothman, 2015; Lucas et al., 2009; Wright et al., 2009; Yamashita et al., 2009). Some foods may be relatively rare and highly nutritious, while other foods may be relatively abundant but low quality (Lambert & Rothman, 2015; Marshall & Wrangham, 2007; Melin, Young, et al., 2014). “Low quality” foods typically have lower energy, a lower protein‐to‐fiber ratio, and/or may contain more potentially toxic plant secondary metabolites (PSMs), like tannins and terpenoids, than “high quality” foods (Doran‐Sheehy et al., 2009; Lambert, 2002; Marshall & Wrangham, 2007). “Low quality” foods are also often more mechanically difficult to access, process, and/or digest than “high quality” foods (Lambert, 1998; McGraw et al., 2014).

Plant pith, the spongy interior of plant stems, is typically considered to be a relatively low‐quality resource and is suggested to be a FBF for some primates (Conklin‐Brittain et al., 1998). Pith is widely and consistently available yet is difficult to access/chew (Yamashita et al., 2009), is highly fibrous (Rothman et al., 2006; Wrangham et al., 1991) and may contain large quantities of PSMs (Huffman, 1997; Theis & Lerdau, 2003). Interestingly, its consumption has been reported in several primate species (Knott, 1998; Matthews et al., 2020; Rothman et al., 2006; Tutin et al., 1997), and at least in some cases can be consumed non‐nutritionally for its medicinal properties (e.g., *Vernonia amygdalina* pith consumption by chimpanzees, Huffman, Gotoh, et al., 1997; Huffman, Gotoh, et al., 1993). In such cases, pith consumption is linked to the presence of various bioactive PSMs, including terpenoids, alkaloids, and tannins (De la Fuente et al., 2022; Koshimizu et al., 1994; Ohigashi et al., 1994). For primates with relatively large jaws and hindguts, including chimpanzees, gorillas, and bonobos, pith fiber offers a viable source of energy during periods of fruit dearth through the provision of hemicellulose, with minimal digestive consequences from the consumption of PSMs (Kano & Mulavwa, 1984; Lambert, 1998; Wrangham et al., 1991). However, pith processing may be more difficult for smaller frugivores with gracile jaws/dentition and simple stomachs (Wright et al., 2009). Because pith consumption requires primates to first strip tough plant stems, accessing it presents the possibility of jaw and tooth damage, and it often contains high concentrations of fiber on a dry weight basis as well as PSMs, potentially inducing digestive challenges (Dominy et al., 2008; Thiery et al., 2017). Given the mechanical and chemical challenges associated with pith foraging, and its potential importance in surviving lean periods, it is surprising that little is known about the factors contributing to its selection. We begin to address this in our present study.

White‐faced capuchins (*Cebus imitator*) of the Central American tropical dry forest are small‐bodied (2.7−3.8 kg; Perry, 1997) frugivore‐omnivores that are reported to occasionally consume plant pith (Melin et al., 2020; Perry et al., 2012). Capuchins have been documented eating the pith of a few tree species including *B. simaruba*, *Trichilia americana*, and *Spondias mombin*, but seemingly ignore the other 300+ tree species in the dry forest, including many abundant species (Mosdossy et al., 2015). Plant pith is mechanically challenging to access, likely at the extreme of gracile capuchin masticatory and digestive ability (Fragaszy et al., 2004; Janson & Boinski, 1992); as such, understanding pith selection has the potential to reveal the range and limits of dietary flexibility in this frugivorous‐omnivorous species (Phillips, 1995; Wright, 2005; Wright et al., 2009). To better understand the patterns of plant pith selection, including disentangling the influences of resource abundance, chemical and mechanical defense, and nutritional content, comprehensive study of behavioral and chemical ecology is needed. Here we present such an integrative analysis.

We assess the timing, patterns, and potential drivers of pith foraging and selection by wild white‐faced capuchin monkeys relative to both ecological variables and the physical and chemical properties of dietary and non‐dietary pith. We first seek to determine when pith is consumed and if its consumption is consistent with the role of a fallback food for capuchin monkeys. We predict that pith foraging will occur seasonally and increase as (a) habitat‐wide fruit abundance and (b) visits to fruit patches both decrease. Second, we explore pith foraging relative to plant properties by investigating if dietary species differ from the most abundant non‐dietary plant species in: relative pith quantity (i.e., pith per branch), mechanical hardness, odor profile, and macronutrient profile. We predict that relative to non‐dietary species, dietary species will: (a) contain relatively more pith per branch; (b) have softer outer bark and pith; (c) differ in their odor profiles, notably by containing fewer potential toxins; and (d) contain more protein and less fiber.

## 2 METHODS

### 2.1 Study site

This study was conducted in Sector Santa Rosa (SSR) in the Área de Conservación Guanacaste, located in northwest Costa Rica. SSR consists of tropical dry forest in various stages of regeneration following intense restoration efforts beginning in the 1970s (Janzen & Hallwachs, 2020). SSR is characterized by two distinct seasons: a hot dry season from December to May, in which almost no rainfall occurs, and a cooler wet season from May to November, in which 900−2300 mm of rain falls (Janzen, 1988). Climatic seasonality is accompanied by fluctuations in resource abundance, including fruits and invertebrates (Hogan & Melin, 2018; Janzen, 1988; Mosdossy et al., 2015). The capuchins at SSR have been studied for 40 years as part of the long‐term Santa Rosa Primate Field Project (Fedigan & Jack, 2012; Melin et al., 2020). Insights from this long‐term research program have revealed that climatic seasonality influences capuchin behavior and ecology in a variety of ways, including seasonal changes in diet (Bergstrom et al., 2019; Melin, Young, et al., 2014), parasite infection properties (Parr et al., 2013a, 2013b), and gut microbiome composition (Orkin et al., 2019). Capuchins are frugivore‐omnivores with diets typically consisting anywhere from 50% to 80% fruit and 15%−45% invertebrates (Fragaszy et al., 2004; Melin et al., 2008). Other plant parts, like flowers and pith, typically occupy between 0% and 8% of the diet (Mosdossy et al., 2015).

### 2.2 Quantifying foraging behavior

Members of the Santa Rosa Primate Field Project followed each group twice per month from the hours of 6:00−12:00. During group follows, we recorded all‐occurrences of “patch visits” every time any member of the group enters a new plant‐based food patch, noting the plant part eaten, the tree species, the location (via GPS point at the tree), the diameter at breast height, and phenology based on an established scoring system (Melin, Hiramatsu, et al., 2014; Melin et al., 2018). To determine the relationship between pith foraging and fruit foraging & abundance, we analyzed a large data set of pith (*n* = 312) and fruit and flower (*n* = 3846 and *n* = 33, respectively) “patch visits” collected between June 2018 and February 2022. We used these pith patch data, in addition to historical data on pith foraging (Melin, 2011; Mosdossy et al., 2015), to choose dietary pith species to sample. A video of pith foraging behavior is available in our Supporting Information: Video S1.

### 2.3 Quantifying habitat wide fruit & tree abundance

For the duration of the study, we collected standardized monthly data on tree phenology throughout the study groups' home ranges, from which we generated monthly estimates of habitat‐wide fruit abundance in kilograms per hectare (for detailed methods see Hogan & Melin, 2018; Orkin et al., 2019). We used this measure to assess foraging behavior in relation to fruit availability. We additionally leveraged an extensive data set of tree transects (>40,000 trees) at SSR (Melin, 2011; Orkin et al., 2019; Owen et al., 2020) to identify the top seven most abundant, but non‐dietary, species to sample. Tree counts were conducted over 273,100 × 4 m transects running north−south, and 151,100 × 2 m transects running east−west; trees were required to be at least 100 cm tall to be counted. In addition, we recorded daily rainfall using a rain gauge at the field station.

### 2.4 Pith sample collection

**Figure 1:**
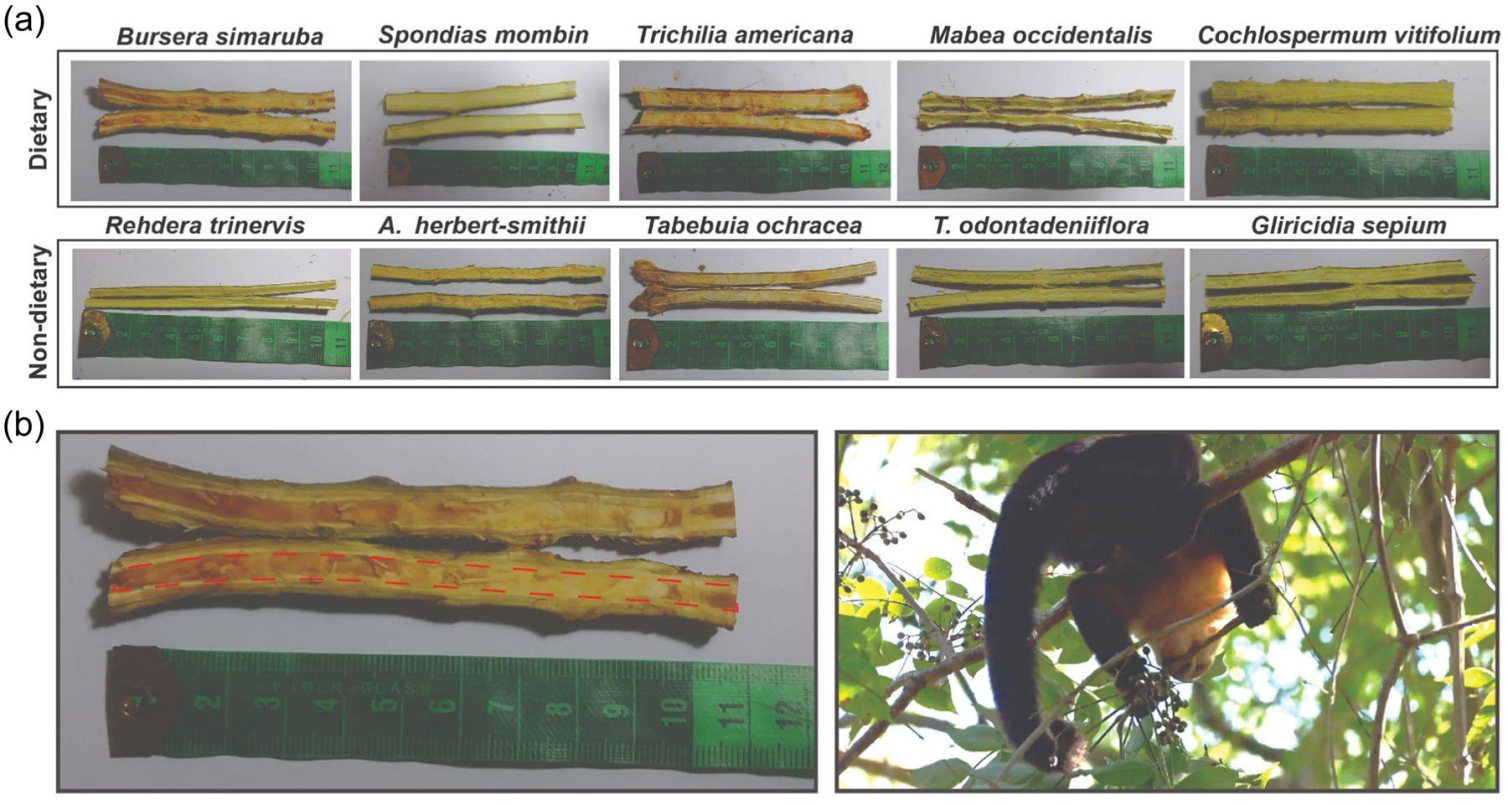
(a) Example dietary and non‐dietary pith species sampled in 2019. Dietary species: *Bursera simaruba*, *Spondias mombin*, *Trichilia americana*, *Mabea occidentalis*, and *Cochlospermum vitifolium*; non‐dietary species: *Rehdera trinervis*, *Ateleia herbert‐smithii*, *Tabebuia ochracea*, *Tabernaemontana odontadeniiflora* (previously *Stemmadenia obovata*), and *Gliricidia sepium*. (b) (left): close up of *B. simaruba* pith, highlighted in red and (b) (right): adult male capuchin feeding on the pith of a young *B. simaruba* branch (Credit: Amanda Melin).

To assess the impact of plant physical and chemical properties on selectivity, we compared relative pith quantity, mechanical hardness, odor composition, and macronutrient profile in a total of five dietary and seven non‐dietary plant species (Supporting Information: Table S1). We sampled two young branches from each of five individual trees per species (*n* = 10 branches per species, 120 branches total) in December 2019. Representative branches were sampled directly from the canopy, using pruning poles as needed. We chose branches to best represent the size and age of the branches selected by capuchins during pith foraging. Example pith species are shown in Figure 1. As described above, we used pith foraging data to choose dietary species, and we used tree abundance data to select the top seven most abundant non‐dietary tree species. Sampling the most abundant, but non‐dietary, pith species allows us to tease apart the influence of plant traits and species abundance.

### 2.5 Relative pith quantity

To determine the relative amount of pith present per species, we trimmed each branch to a length of 10 cm and then bisected the branch longitudinally. Plant pith is easy to discern from the surrounding xylem and phloem due to its unique texture and appearance. We photographed each branch against a scale to measure pith quantity using the software ImageJ (Figure 1). In this program, we measured the width of the pith at three different locations along each branch. We then divided the width of the pith at these locations by the total width of the branch to obtain a measure of relative pith quantity (i.e., the proportion of the branch occupied by pith). We used the average of these three measurements in our analysis.

### 2.6 Mechanical hardness

We measured indentation hardness of each branch using the sharp indent test from the Lucas FLS‐1 mechanical tester. This measured resistance to deformation in gigapascals (gPa) using a Vicker's pyramid (Lucas et al., 2009). We first tested the repeatability and consistency of the mechanical tester by measuring a single branch multiple times and confirming that the hardness values are consistent across measurements. Given the high level of repeatability, for our main data set we measured each branch once. To test the mechanical hardness of both the outer and inner branch surfaces, we first placed the bisected branch face down and tested the hardness of the outside of the branch (i.e., the outer bark; *n* = 120) at approximately 5 cm, midway down the branch. We then flipped the branch so that the pith was facing up and proceeded to test the pith itself in the same way (*n* = 120). We obtained one hardness value per surface per branch, for a total of 240 hardness measurements.

### 2.7 Odor sampling and analysis

We characterized pith odor profile by quantifying volatile organic compound (VOC) content in terms of richness (i.e., the number of VOCs detected) and composition (i.e., presence/absence and relative abundance of VOCs). Odor can directly inform chemical composition and thus may be used by capuchins during foraging to assess pith edibility and nutritional content (Nevo & Heymann, 2015; Nevo et al., 2019; Schmitt et al., 2018). For odor sampling, we removed and pooled the pith from each individual branch per species, resulting in 5 samples per species. We were able to sample 7 out of 12 species (Supporting Information: Table S1; *n* = 35 total odor samples). The piths of the remaining species were too small and/or mechanically difficult to remove, rendering us unable to isolate the pith and extract its odor profile. We sampled pith VOCs using chromatoprobe VOC traps and an established on‐site semi‐static headspace procedure (Nevo & Valenta, 2018). We then generated thermal desorption–gas chromatography‐mass spectrometry (GC‐MS) profiles at Ulm University, Germany. Two vials containing odor samples (one *S. mombin* and one *Tabernaemontana odontadeniiflora*) opened during transportation and had to be discarded, resulting in a final data set of *n* = 33 for GC‐MS analyses. We processed the GC‐MS data using a semiautomatic procedure following Weiß et al. (2018) and identified VOCs using the National Institute of Standards and Technology mass spectral library (NIST14, Shen et al., 2014). Detailed information on pith odor sampling, GC‐MS analyses, GC‐MS data interpretation, and VOC identification is provided in Supporting Information: Table S2. Following Knudsen et al. (2006), we grouped all VOCs into five distinct chemical classes (i.e., aliphatics, benzenoids and phenylpropanoids, terpenoids, nitrogen‐containing compounds, and miscellaneous cyclic compounds), plus an “unidentified” group. Of the 122 VOCs selected during GC‐MS data processing, 112 (92%) were assigned one of the five chemical classes, of which 79% were identified with reasonable confidence (Supporting Information: Table S3).

### 2.8 Nutritional sampling and analysis

To measure the macronutrient profile of dietary and non‐dietary pith samples, we removed the pith from each branch and pooled it by species to achieve sufficient dry mass for nutritional assays (30 g; Rothman et al., 2012). We were able to gather sufficient dry mass to analyze 7 of our 12 species (Supporting Information: Table S1). We used a Nesco Gardenmaster to dry our pith samples at 45°C, having weighed our samples pre‐ and post‐drying. We sent our pith samples to Dairy One Laboratories in Ithaca, NY, where they were analyzed for crude protein, available protein, crude fat, water soluble carbohydrate, fiber, and ash content using established assays (Rothman et al., 2012).

### 2.9 Statistical analyses

#### 2.9.1 Ecological variables: When is pith consumed, and is it a fallback food?

To determine whether fruit abundance and/or fruit foraging predict pith consumption, we built a negative binomial generalized linear model with monthly pith patch visit count as the response variable using the glm.nb function from the *MASS* R package (Venables & Ripley, 2002). We included monthly fruit biomass, monthly fruit patch visits, and monthly rainfall (in mm) as predictor variables. To control for observation time per month, we include an offset variable of monthly contact time in hours (model formula: glm.nb(MonthlyPithPatchVisits − MonthlyFruitBiomass + MonthlyFruitPatchVisits + MonthlyRainfall + offset(log(MonthlyContactTime)). To assess the seasonality of pith patch visits, we used the R package mgcv to build a generalized additive model with monthly pith patch count as the response variable and a cyclical spline to model month as a predictor variable (Wood, 2011). We chose this method to account for the circular nature of time series data while controlling for contact time. Our model formula was as follows: gam(MonthlyPithPatchVisits − offset(log(MonthlyContactTime)) + s(Month, bs = “cc,” *k* = 12), method = “REML,” family = poisson(link = “log”)). For both models, our analysis was conducted at the level of month per year (*n* = 45 months). All analyses were conducted in R version 4.2.2 (R Core Team, 2022).

#### 2.9.2 Plant properties: Do dietary species differ from non‐dietary species in relative pith quantity, hardness, odor profile, and/or macronutrient profile?

To test for differences between dietary and non‐dietary pith species on average pith quantity, mechanical hardness, sample odor richness (i.e., the number of VOCs detected), and macronutrient concentrations, we conducted multiple independent Wilcoxon rank‐sum tests from the *stats* R package to test if dietary and non‐dietary species differ in these four variables. The Wilcoxon rank‐sum test compares two paired groups (i.e., dietary vs. non‐dietary species) to determine if they differ significantly from one another. The sample sizes contributing to our data analyses for each variable are summarized in Table 1. For mechanical hardness, we analyzed the outer bark and the pith separately. We further tested for differences in pith sample odor composition, accounting for both presence/absence and relative abundance of VOCs, using a nonparametric analysis of similarity (ANOSIM, using 999 permutations) from the *vegan* R package (Oksanen et al., 2023). ANOSIM was based on the Bray−Curtis dissimilarity indices calculated from the VOCs relative peak areas (i.e., peak area divided by the sum of all included peak areas x 100) in each sample, to account for variation in absolute abundance due to varying amount of pith collected.

**Table 1:** Summary of sample sizes for our pith analyses.

Measurement	Number of species	Number of observations in analysis
Pith patch visits	11 dietary species recorded over 45 months	312 pith patch visits over 45 months
Relative pith quantity	Pith quantity measured from 12 species (5 dietary, 7 non‐dietary)	120 measurements (10 branches per species)
Mechanical hardness	Pith hardness measured from 12 species (5 dietary, 7 non‐dietary)	240 measurements from 120 samples (120 outer branch, 120 pith, 10 branches per species)
Odor composition	Pith odor composition measured from 7 species (4 dietary, 3 non‐dietary)	33 odor samples (4−5 samples per species)^a^
Macronutritional profile	Pith macronutrient profile measured from 7 species (4 dietary, 3 non‐dietary)	7 samples (1 per species)

*Note*: See Supporting Information: Table S1 for details about which species were sampled for each of the different measurement types.

^a^ One *Spondias mombin* and one *Tabernaemontana odontadeniiflora* odor samples degraded during transportation and were removed from the data set.

## 3 RESULTS

### 3.1 Ecological variables: When is pith consumed, and is it a fallback food?

**Figure 2:**
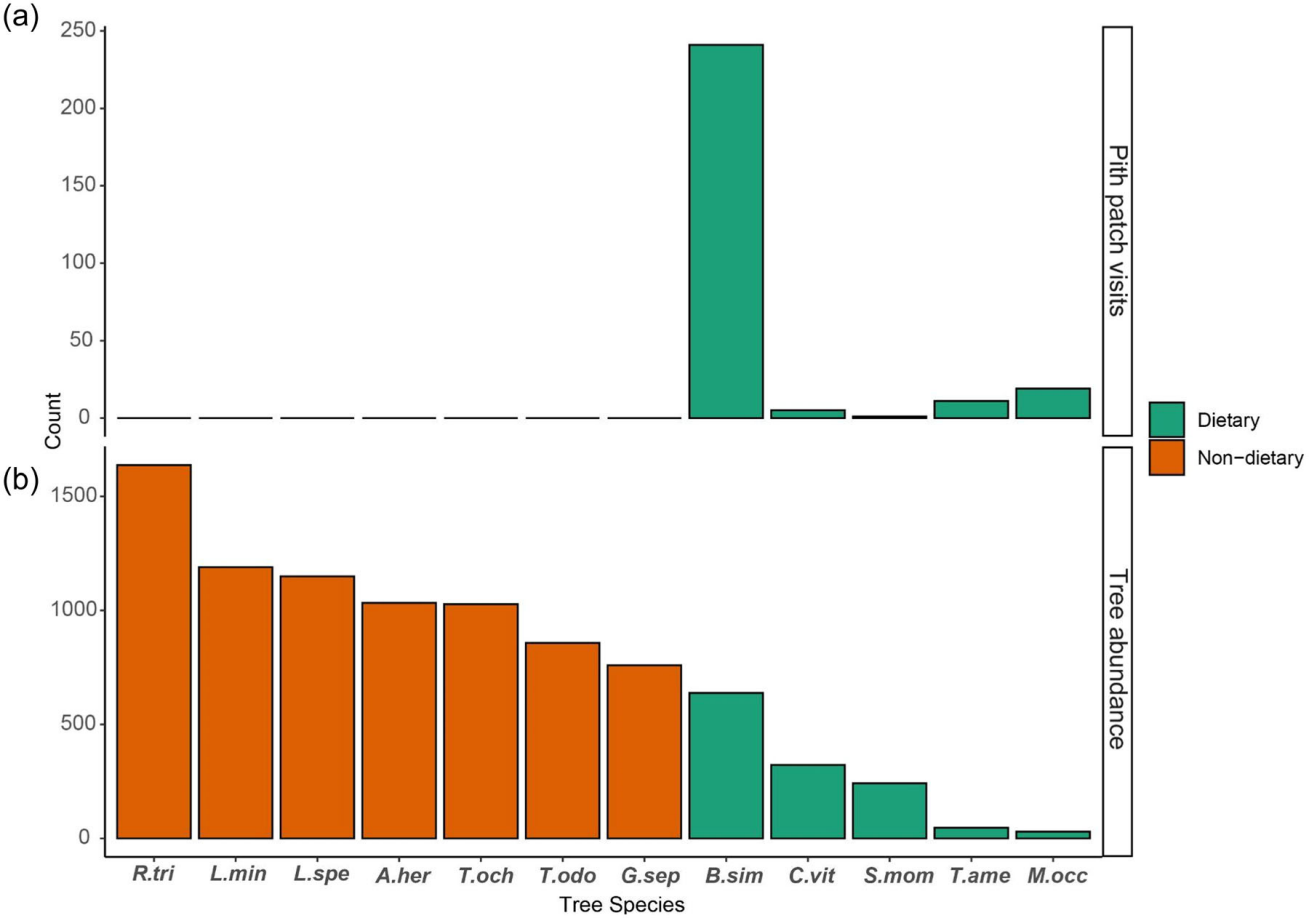
Pith patch visits and tree counts. (a) number of pith patch visits for species analyzed in this study spanning the period June 2018 to February 2022. (b) Tree counts for species analyzed in this study based on transect surveys. Species codes are as follows: A.her, *Ateleia herbert‐smithii*; B.sim, *Bursera simaruba*. C.vit, *Cochlospermum vitifolium*; G.sep, *Gliricidia sepium*; L.min, *Lonchocarpus miniflorus*; L.spe, *Luehea speciosa*; M.occ, *Mabea occidentalis*; R.tri, *Rehdera trinervis*; S.mom, *Spondias mombin*; T.ame, *Trichilia americana*; T.och, *Tabebuia ochracea*; T.odo, *Tabernaemontana odontadeniiflora*.

**Figure 3:**
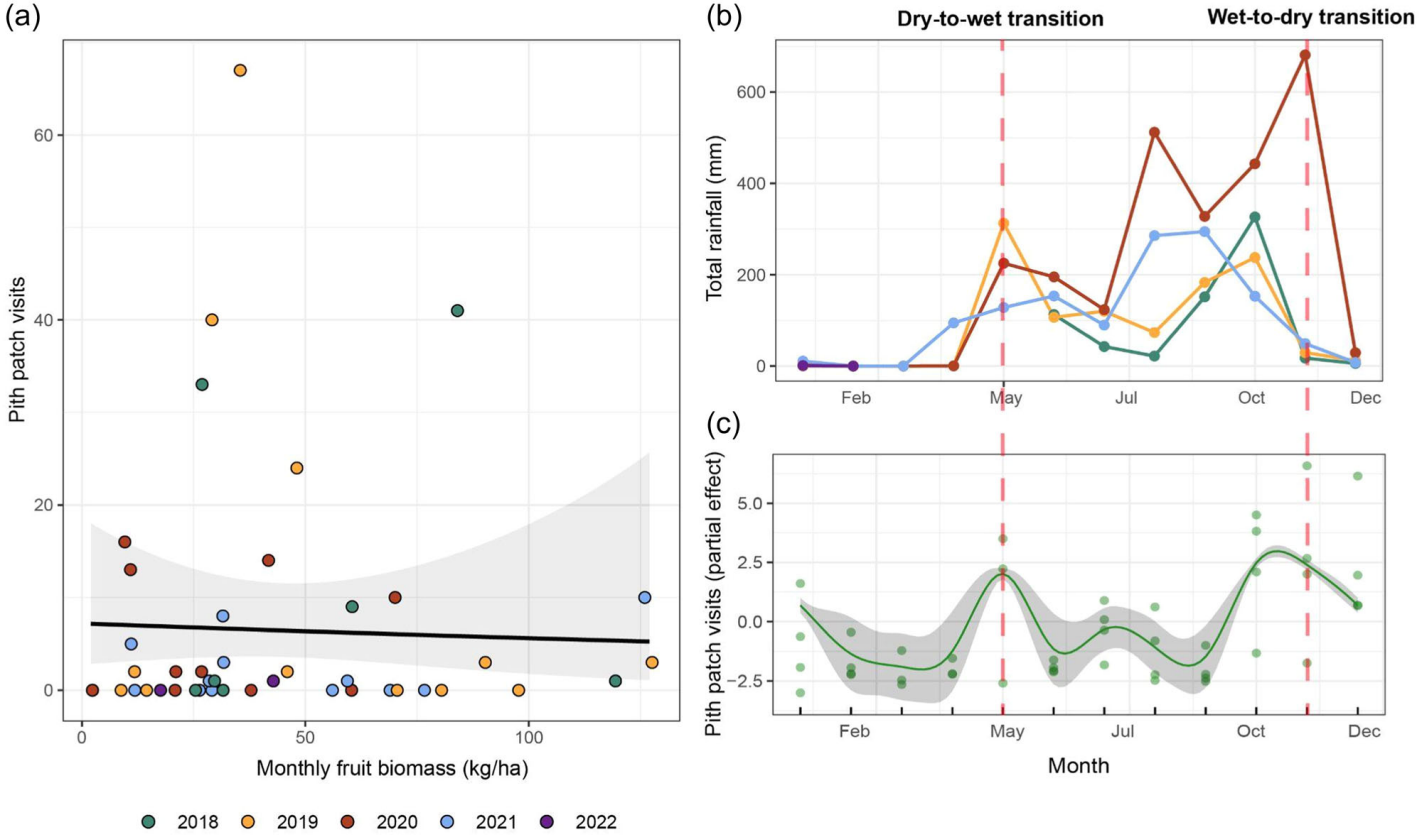
Results of pith patch visit analysis spanning June 2018 to February 2022. (a) Negative binomial regression of pith patch visits against fruit biomass in kg/ha color‐coded by year. (b) Monthly rainfall between June 2018 and February 2022 color‐coded by year. (c) Generalized additive model of pith patch visits as a function of month; points denote partial model residuals.

From June 2018 to February 2022, we recorded 312 pith patch visits to 11 unique species (Supporting Information: Table S4). For dietary context, we recorded 3846 fruit patch visits, and 33 flower visits, during this same period; pith visits made up 7% of the total vegetative patch visits. The vast majority of pith patch visits (~77%) were of the species *B. simaruba*, which was the most abundant dietary pith species in our analysis (Figure 2a,b), despite the fact that it was not the most abundant species at SSR (Figure 2b). We found no significant effect of either fruit variable, nor a significant effect of rainfall (Figure 3 and Table 2). We did, however, find a significant effect of month in our generalized additive model (Table 2), suggesting seasonality in pith patch visits, with the vast majority (84%) occurring around seasonal transitions, at the beginning of the wet season in May (77 PPVs), and the end of the wet season in October (104 PPVs) and November (81 PPVs; Figure 3).

**Table 2:** Results of a negative binomial model assessing pith patch visits in relation to fruit variables and rainfall, and a generalized additive model assessing pith patch visits in relation to month.

Model term	Estimate	SE	*Z* value	*p* Value
Generalized linear model (negative binomial)				
Intercept	−2.9597	0.5929	−4.992	<0.001*
Fruit patch visits	−0.0009	0.0028	−0.325	0.745
Fruit biomass	−0.0025	0.0089	−0.276	0.783
Rainfall	0.0026	0.0018	1.387	0.165
Generalized additive model (Poisson)				
Intercept	−3.9402	0.1617	−24.37	<0.001*

**Smooth term**	**edf**	**Ref. *df***	***χ* ^2^**	***p* Value**
s (month)	8.663	10	243.9	<0.001*

Abbreviation: SE, standard error.

*p < 0.05.

### 3.2 Plant properties: Do dietary species differ from non‐dietary species in relative pith quantity, hardness, odor profile, and/or macronutrient profile?

**Figure 4:**
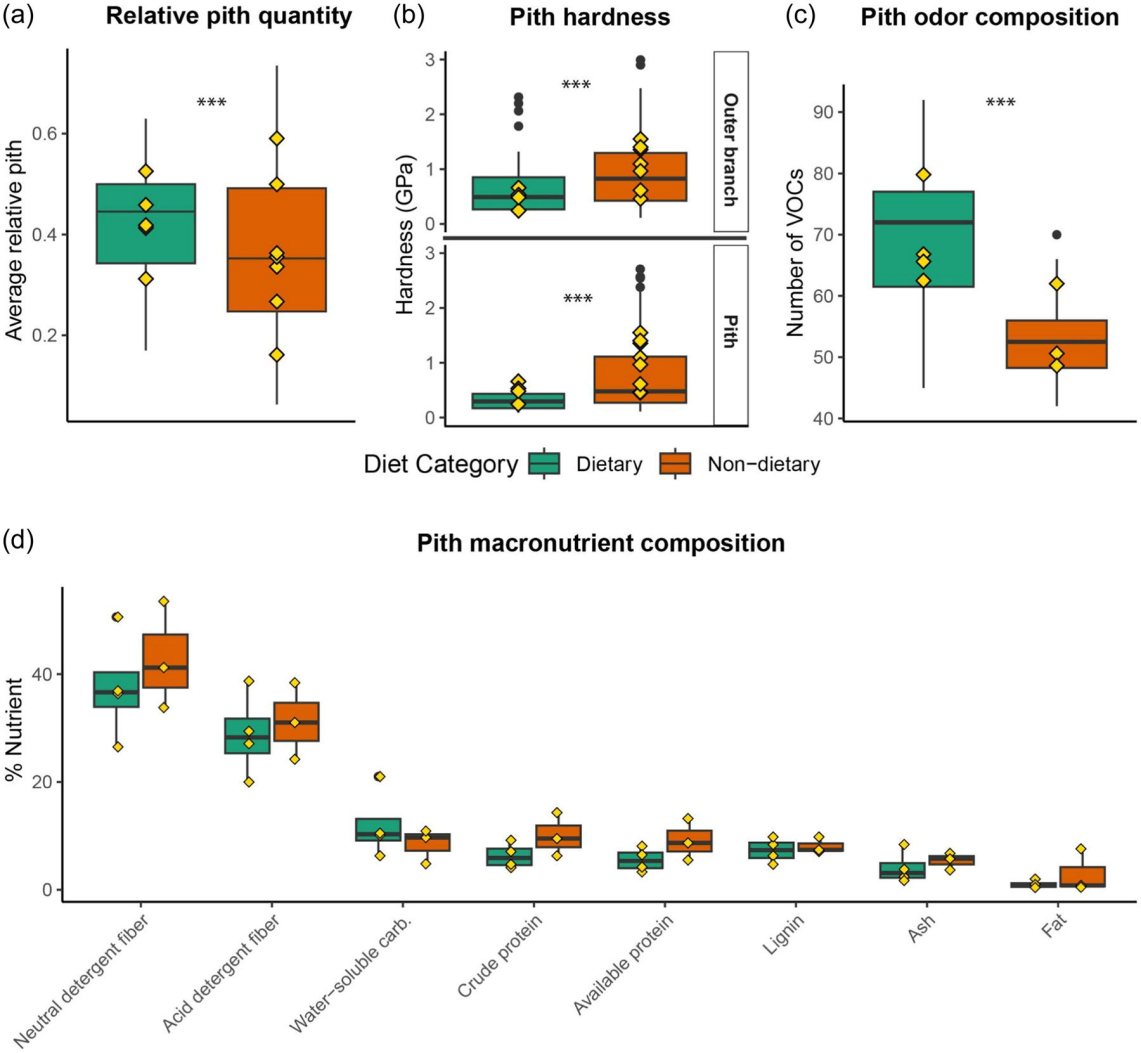
Boxplots comparing dietary and non‐dietary pith species in the following variables: (a) average relative pith quantity; (b) mechanical hardness of outer bark and pith; (c) number of volatile organic compounds (VOCs); and (d) macronutritional profile. Yellow diamonds denote individual species means; asterisks denote statistical significance (*p* < 0.05) for Wilcoxon rank‐sum tests comparing dietary versus non‐dietary species.

**Figure 5:**
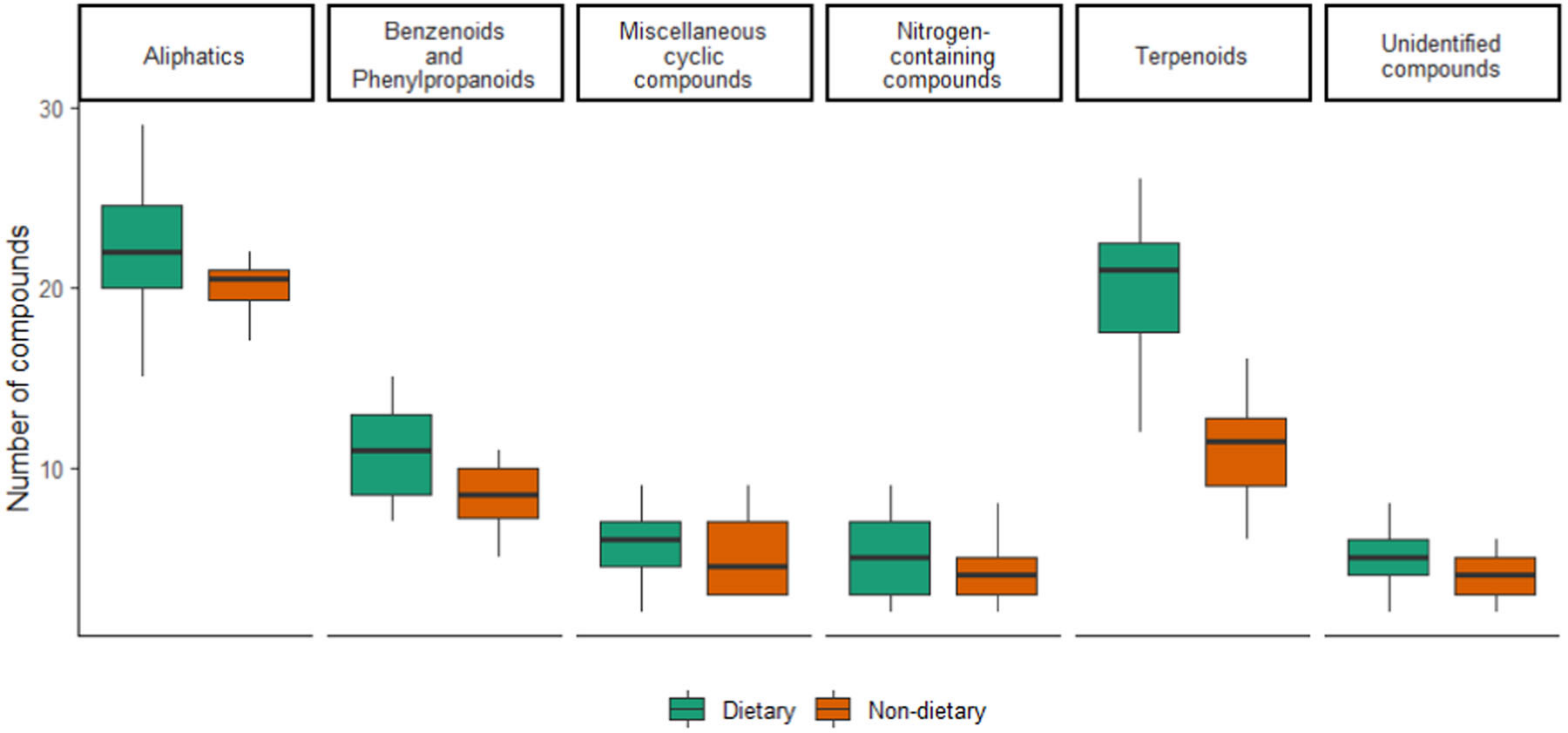
Prevalence of six different VOC classes retrieved from the pith of dietary and non‐dietary species. VOC, volatile organic compound.

We found that dietary pith species contained relatively more pith per branch on average (*W* = 2193, *p* = 0.021; Figure 4a). We further found that dietary pith species were softer (i.e., lower indentation hardness value) than non‐dietary pith species. This was found both for the outer bark (*W* = 1225, *p* = 0.005), and the pith (*W* = 868, *p* < 0.001; Figure 4b). Dietary pith odor richness was greater than that of non‐dietary species (*W* = 231.5, *p* < 0.001; Figure 4c), and odor composition differed significantly between dietary and non‐dietary species (ANOSIM: *R* = 0.36, *p* = 0.001). When considering six broad chemical categories of VOCs retrieved from the samples (i.e., aliphatics, benzenoids and phenylpropanoids, terpenoids, nitrogen‐containing compounds, miscellaneous cyclic compounds, and unidentified compounds), the relative proportion of chemical classes found in both dietary and non‐dietary pith were in the same ranges, with dietary pith containing more terpenoids (mean ± SD = 19.7 ± 4.2) than non‐dietary pith (mean ± SD = 10.9 ± 2.7; Figure 5). Identities of these terpenoids and their individual abundances in the different samples are shown in Supporting Information: Figure S1. We were able to identify three terpenoids, ⍺‐terpinene, camphene, and caryophyllenyl alcohol, only occurring in dietary pith species (Supporting Information: Figure S1). Lastly, we found no significant differences between dietary and non‐dietary pith for any of the macronutrients tested (Figure 4d).

## 4 DISCUSSION

In this study we sought to comprehensively assess the timing, patterns, and drivers of plant pith selection by wild white‐faced capuchin monkeys and determine if pith is a FBF for capuchins. Using a large, multiyear data set of pith patch visits and a novel multicomponent analysis of dietary and non‐dietary piths, we find that pith consumption is seasonal and highly selective. Rather than correlating with fruit abundance and/or fruit patch visits, pith patch visits instead cluster around months of seasonal transition, particularly at the beginning and end of the wet season. We further find that relative pith quantity, mechanical hardness, and odor profile may contribute to pith selectivity, with dietary pith species containing relatively more pith per branch, exhibiting softer branches both inside and out, and containing more VOCs, particularly terpenoids.

### 4.1 Ecological variables: When is pith consumed, and is it a fallback food?

Given that plant pith is suggested to be a fallback food across primate taxa (Lambert & Rothman, 2015), we predicted that capuchins consume pith when fruit is scarce, and thus that pith patch visits would negatively correlate with habitat‐wide fruit abundance and/or fruit patch visits. Contrary to our prediction, we find no relationship between monthly number of pith patch visits and monthly habitat‐wide fruit abundance or fruit patch visits. This suggests that pith is not a fallback food in the classic sense (Alberts et al., 2005; Marshall et al., 2009). In this respect they seem to differ from other non‐fruit foods, such as embedded invertebrates, which have been argued to serve as a FBF for white‐faced capuchins and follow the more typical pattern of higher consumption when fruit is scarce (Melin, Young, et al., 2014). Patterns of plant pith consumption indicate that pith may be more consistent with the role of a seasonal dietary supplement, consumed to provide either key nutrients and/or medicinal PSMs that may be otherwise missing from their diet (DiGiorgio et al., 2023; Lambert & Rothman, 2015). We find that pith patch visits cluster around seasonal transitions, from the dry season to the wet season and vice versa, with no clear relationship to fruit variables. Given that pith is likely to be equally abundant year‐round, this raises the question as to why capuchins show this pattern with respect to seasonal transitions. One possible explanation is plant medicinal properties.

Seasonal transitions in Santa Rosa are a period of intense environmental change characterized by rapidly shifting temperatures, precipitation, and fluctuating resources (Campos et al., 2015; Janzen, 1993; Orkin et al., 2019). These transitions can also be accompanied by particularly intense shifts in parasite load and gut microbiome composition (Orkin et al., 2019; Parr et al., 2013a, 2013b), raising the possibility that pith consumption may be a response to seasonally shifting health‐related variables. Seasonal pith consumption of certain bioactive species has been documented in some primates (De la Fuente et al., 2022; Huffman, 2017). Chimpanzees, for example, are known to consume the bitter pith of *Vernonia* plants in response to seasonal nematode infection (Huffman, Gotoh, et al., 1997; Ohigashi et al., 1994; Rodriguez & Wrangham, 1993). There are intriguing parallels between pith consumption by capuchins and by chimpanzees. Like capuchins, chimpanzees are highly omnivorous, large‐brained primates, who also exhibit highly seasonal and selective pith consumption (Huffman, 1997; Koshimizu et al., 1994; Wrangham et al., 1991). Omnivory is often linked to parasitism, as animal prey are often secondary hosts in parasite life cycles, which may necessitate seasonal usage of medicinal plants (Hernandez et al., 2009; Huffman, Gotoh, et al., 1997; Singer & Bernays, 2003; Springer & Kappeler, 2016). Future studies should seek to investigate the correlation between pith foraging and internal variables such as parasite load and gut microbiome composition.

### 4.2 Plant properties: Do dietary species differ from non‐dietary species in relative pith quantity, hardness, odor profile, and/or macronutrient profile?

In line with our predictions, we find that relative pith quantity, mechanical hardness, and odor composition may all contribute to pith selectivity. We find that dietary species have more pith per branch, are softer inside and out, and have a distinct odor profile (i.e., containing more VOCs and a different assemblage of them). Contrary to our nutrition prediction, we find that dietary and non‐dietary piths do not differ in their macronutritional profiles, suggesting that macronutritive value is not a primary driver of pith selection. The piths in our study contain approximately 10% water soluble carbohydrates and 39% fiber, indicating that they are less sugary and more fibrous on average than fruits in Santa Rosa, as previous studies have reported fruits from our field site to contain 38% water soluble carbohydrates and 30% fiber (Bergstrom et al., 2018). Taken together, our results suggest that capuchins eat and ostensibly prefer the pith of species that have a greater relative quantity of pith per branch, as eating pithier species increases the reward per unit time of effort required to access pith. Furthermore, dietary pith species exhibit significantly lower hardness, indicating that dietary piths are softer, and thus easier to access and chew. By preferentially selecting species that are softer than others, these gracile capuchins can reduce the mechanical challenge of pith foraging, and thus minimize the possibility of jaw and tooth damage, while maximizing the benefit of pith consumption (Reed & Ross, 2010; Rosenberger, 1992; Thiery et al., 2017). Robust capuchins (genus *Sapajus*) have been reported to consume hard and tough foods, including occasionally consuming palm pith (Terborgh, 1983; Wright et al., 2009). Unfortunately, mechanical hardness data of palm piths are not available for direct comparison. However, we would expect that the range of piths consumed by robust capuchins could include significantly harder foods than those consumed by the gracile capuchins, as *Sapajus* have more robust teeth and jaws (Alfaro et al., 2012; Wright, 2005; Wright et al., 2009). These physical variables—pith quantity and hardness—help explain why some species are chosen over others but cannot explain the underlying motivation behind pith consumption. Our odor results begin to shed light on this question.

Dietary piths contain significantly more VOCs than non‐dietary piths, and the two categories differ in their odor profile. The class of compounds primarily responsible for the differences observed between dietary and non‐dietary pith is that of terpenoids, a class of bioactive compounds known for their diverse medicinal properties (Cox‐Georgian et al., 2019; Kandi et al., 2015). Coupled with the finding that pith consumption occurs primarily during seasonal transitions, this suggests that pith could be consumed for medicinal properties in response to seasonally shifting health variables (Mosdossy et al., 2015). Capuchins prefer to consume the pith of *B. simaruba* (>77% of pith patch visits), known locally as gumbo limbo. *B. simaruba* is well‐known in traditional medicine practices for its diverse medicinal uses, with various parts exhibiting anti‐inflammatory, antimicrobial, and antioxidant properties (Bah et al., 2014; Camporese et al., 2003; Preethi et al., 2020; Sosa et al., 2002). *B. simaruba* is particularly valued by practitioners of traditional medicine for its highly fragrant, bioactive resin (also called “copal”), which is produced in high quantities by stems and branches in response to damage (Murthy et al., 2016; Peraza‐Sánchez & Peña‐Rodríguez, 1992; Preethi et al., 2020). As such, capuchins are likely exposed to high quantities of this bioactive resin during pith foraging, which may ultimately drive pith consumption. The stem resin of *B. simaruba* and that of its congeners has widely known and well‐demonstrated anti‐inflammatory and antimicrobial properties, some of which are linked to terpenoid profile (Blancas et al., 2022; Flores & Ricalde, 1996; Gigliarelli et al., 2015; Junor et al., 2007; Romero‐Estrada et al., 2016). Future studies should seek to examine the bioactive properties of the resiniferous *B. simaruba* pith in more detail, with particular attention to the effects of *B. simaruba* pith on the gastrointestinal helminths that parasitize capuchins (Parr et al., 2013a, 2013b).

### 4.3 Limitations and future directions

It is important to also recognize the limitations of our study. First, our analysis of plant properties had a limited sample of non‐dietary species. The pith of many non‐dietary species was too difficult to access and remove, and thus could not be analyzed for their odor and macronutritional profiles. This indicates that the data reported are conservative, and further supports the conclusion that dietary species are selected for being relatively soft. Further, we only collected pith samples during a single month, and there may be more variation in pith traits than our snapshot suggests. Future work would benefit from assessing pith traits over time, especially in different seasons, to capture potential variability. Lastly, our “patch visit” approach does not record intake rates or relative amounts of pith foraging by adults and by juveniles, which may obfuscate variation in the nutrient intake rate of pith versus other food types, and in the dietary importance of pith to different group members. These would be promising areas for future research. Importantly, regarding the latter point, it is relevant to note that pith foraging is highly synchronous, with many individuals of all age classes foraging simultaneously, that is, pith foraging does not seem to be merely juvenile “experimentation” (Eadie, 2015). Finally, to more fully examine the role of pith in the capuchin diet, future work could aim to analyze relevant fruit traits, like the toughness and hardness of the husks of mechanically protected species, in relation to pith traits to better understand the full range of mechanical challenges faced by capuchins. Such an analysis would allow further elucidation of where pith fits into the broader picture of capuchin foraging ecology.

## 5 CONCLUSIONS

Our study is the first to provide a comprehensive examination of multiple factors that may shape the patterns and drivers of plant pith selection by wild primates. Rather than eating pith when their preferred food—ripe fruit—is scarce, we find instead that capuchin monkeys consume pith primarily during seasonal transitions, suggesting a relationship between seasonality and pith foraging. Our results do not support the role of pith as a fallback food for white‐faced capuchins. Rather, pith likely fulfills the role of a seasonally important dietary supplement. Further, pith selection is a complex, multifaceted process, in which dietary species may be chosen for a combination of their relative pith quantity, mechanical hardness, and their odor composition. Our results further suggest a medicinal role of pith consumption, and future studies should seek to explicitly test this possibility through correlational studies with parasite load and in vitro assays, as well as conduct more advanced chemical analyses, including micronutrient and non‐volatile chemical analyses, with a larger sample size. In sum, this study sheds new light on the complex processes underlying resource use by an omnivorous primate.

## AUTHOR CONTRIBUTIONS

**Allegra N. DePasquale**: Conceptualization (lead); data curation (lead); formal analysis (lead); funding acquisition (equal); methodology (equal); project administration (lead); writing—original draft (lead); writing—review and editing (equal). **Alice C. Poirier**: Data curation (equal); formal analysis (equal); methodology (equal); writing—original draft (equal); writing—review and editing (equal). **Megan A. Mah**: Data curation (equal); methodology (supporting); project administration (supporting); writing—review and editing (supporting). **Cinthia Villalobos Suarez**: Data curation (supporting); methodology (supporting); project administration (supporting); writing—review and editing (supporting). **Adrian Guadamuz**: Methodology (equal); writing—review and editing (supporting). **Saul Cheves Hernandez**: Data curation (equal); project administration (supporting); writing—review and editing (supporting). **Ronald Lopez Navarro**: Data curation (equal); project administration (supporting); writing—review and editing (supporting). **Jeremy D. Hogan**: Data curation (supporting); formal analysis (supporting); writing—review and editing (supporting). **Jessica M. Rothman**: Methodology (equal); resources (equal); writing—review and editing (supporting). **Omer Nevo**: Methodology (equal); resources (equal); supervision (supporting); writing—review and editing (supporting). **Amanda D. Melin**: Conceptualization (equal); formal analysis (supporting); funding acquisition (lead); methodology (equal); project administration (equal); resources (equal); supervision (lead); writing—original draft (equal); writing—review and editing (equal).

## CONFLICT OF INTEREST STATEMENT

The authors declare no conflict of interest.

## ETHICS STATEMENT

This research adhered to the laws of Costa Rica and Canada and complied with protocols approved by the ACG (R‐SINAC‐ACG‐PI‐027‐18) (R‐025‐2014‐OT‐CONAGEBIO) and by the Canada Research Council for Animal Care through the University of Calgary's Life and Environmental Care Committee (AC19‐0167).

## Supporting information

Adult male white‐faced capuchin monkey feeding on *Bursera simaruba* pith.

**Suppl. Fig. S1**. Proportion of terpenoid VOCs in the pith samples. The sample size for each species was n = 5, except for *S. mombin* and *T. odontadeniiflora* (n = 4).

Supporting information.

## Data Availability

Data and code for all analyses are available at https://github.com/allegradepasquale/PithAnalysis.git.

## References

[ajp23549-bib-0001] Alberts, S. C. , Hollister‐Smith, J. A. , Mututua, R. S. , Sayialel, S. N. , Muruthi, P. M. , Warutere, J. K. , & Altmann, J. (2005). Seasonality and long‐term change in a savanna environment. In C. P. van Schaik & D. K. Brockman (Eds.), Seasonality in primates: Studies of living and extinct human and non‐human primates (pp. 157–196). Cambridge University Press. 10.1017/CBO9780511542343.007

[ajp23549-bib-0002] Alfaro, J. W. L. , Silva, Jr., J. D. S. E. , & Rylands, A. B. (2012). How different are robust and gracile capuchin monkeys? An argument for the use of *Sapajus* and *Cebus*: *Sapajus* and *Cebus* . American Journal of Primatology, 74(4), 273–286. 10.1002/ajp.22007 22328205

[ajp23549-bib-0003] Altmann, S. A. (2009). Fallback foods, eclectic omnivores, and the packaging problem. American Journal of Physical Anthropology, 140(4), 615–629. 10.1002/ajpa.21097 19890853

[ajp23549-bib-0004] Bah, M. , Gutiérrez‐Avella, D. M. , Mendoza, S. , Rodríguez‐López, V. , & Castañeda‐Moreno, R. (2014). Chemical constituents and antioxidant activity of extracts obtained from branch bark of *Bursera simaruba*.

[ajp23549-bib-0005] Bergstrom, M. L. , Hogan, J. D. , Melin, A. D. , & Fedigan, L. M. (2019). The nutritional importance of invertebrates to female *Cebus capucinus* imitator in a highly seasonal tropical dry forest, American Journal of Physical Anthropology, 170(2), 207–216. 10.1002/ajpa.23913 31396949

[ajp23549-bib-0105] Bergstrom, M. L. , Melin, A. D. , Myers, M. S. , & Fedigan, L. M . (2018). Dietary profile, food composition, and nutritional intake of female white-faced capuchins. In U. Kalbitzer & K. M. Jack (Eds.), Primate Life Histories, Sex Roles, and Adaptability: Essays in Honour of Linda M. Fedigan (pp. 213–243). Springer International Publishing. 10.1007/978-3-319-98285-4_11

[ajp23549-bib-0006] Blancas, J. , Abad‐Fitz, I. , Beltrán‐Rodríguez, L. , Cristians, S. , Rangel‐Landa, S. , Casas, A. , Torres‐García, I. , & Sierra‐Huelsz, J. A. (2022). Chemistry, biological activities, and uses of copal resin (*Bursera* spp.) in Mexico. In H. N. Murthy (Ed.), Gums, resins and latexes of plant origin: Chemistry, biological activities and uses (pp. 433–446). Springer International Publishing. 10.1007/978-3-030-91378-6_21

[ajp23549-bib-0007] Camporese, A. , Balick, M. J. , Arvigo, R. , Esposito, R. G. , Morsellino, N. , Simone, F. D. , & Tubaro, A. (2003). Screening of anti‐bacterial activity of medicinal plants from Belize (Central America). Journal of Ethnopharmacology, 87(1), 103–107. 10.1016/S0378-8741(03)00115-6 12787962

[ajp23549-bib-0008] Campos, F. A. , & Fedigan, L. M. (2009). Behavioral adaptations to heat stress and water scarcity in white‐faced capuchins (*Cebus capucinus*) in Santa Rosa National Park, Costa Rica. American Journal of Physical Anthropology, 138(1), 101–111. 10.1002/ajpa.20908 18711741

[ajp23549-bib-0009] Campos, F. A. , Jack, K. M. , & Fedigan, L. M. (2015). Climate oscillations and conservation measures regulate white‐faced capuchin population growth and demography in a regenerating tropical dry forest in Costa Rica. Biological Conservation, 186, 204–213. 10.1016/j.biocon.2015.03.017

[ajp23549-bib-0010] Chapman, C. A. , Chapman, L. J. , & Wrangham, R. W. (1995). Ecological constraints on group size: An analysis of spider monkey and chimpanzee subgroups. Behavioral Ecology and Sociobiology, 36(1), 59–70. 10.1007/BF00175729

[ajp23549-bib-0011] Chaves, Ó. M. , Fortes, V. B. , Hass, G. P. , Azevedo, R. B. , Stoner, K. E. , & Bicca‐Marques, J. C. (2021). Flower consumption, ambient temperature and rainfall modulate drinking behavior in a folivorous‐frugivorous arboreal mammal. PLoS One, 16(2), e0236974. 10.1371/journal.pone.0236974 33606693 PMC7894884

[ajp23549-bib-0012] Conklin‐Brittain, N. L. , Wrangham, R. W. , & Hunt, K. D. (1998). Dietary response of chimpanzees and cercopithecines to seasonal variation in fruit abundance. II. Macronutrients. International Journal of Primatology, 19(6), 971–998. 10.1023/A:1020370119096

[ajp23549-bib-0013] Constantino, P. J. , & Wright, B. W. (2009). The importance of fallback foods in primate ecology and evolution. American Journal of Physical Anthropology, 140(4), 599–602. 10.1002/ajpa.20978 19890867

[ajp23549-bib-0014] Cox‐Georgian, D. , Ramadoss, N. , Dona, C. , & Basu, C. (2019). Therapeutic and medicinal uses of terpenes. In N. Joshee , S. A. Dhekney & P. Parajuli , (Eds.), *Me*dicin*a*l plant*s: F*ro*m* farm to *pharmacy* (pp. 333–359). Springer International Publishing. 10.1007/978-3-030-31269-5_15

[ajp23549-bib-0015] DiGiorgio, A. L. , Ma, Y. , Upton, E. M. , Gopal, S. , Robinson, N. J. , Susanto, T. , & Knott, C. D. (2023). Famished frugivores or choosy consumers: A generalist frugivore (Wild Bornean Orangutans, *Pongo pygmaeus* wurmbii) leaves available fruit for nonfruit foods. International Journal of Primatology, 44(2), 377–398. 10.1007/s10764-022-00347-2

[ajp23549-bib-0016] Dominy, N. J. , Vogel, E. R. , Yeakel, J. D. , Constantino, P. , & Lucas, P. W. (2008). Mechanical properties of plant underground storage organs and implications for dietary models of early hominins. Evolutionary Biology, 35(3), 159–175. 10.1007/s11692-008-9026-7

[ajp23549-bib-0017] Doran‐Sheehy, D. , Mongo, P. , Lodwick, J. , & Conklin‐Brittain, N. L. (2009). Male and female western gorilla diet: Preferred foods, use of fallback resources, and implications for ape versus old world monkey foraging strategies. American Journal of Physical Anthropology, 140(4), 727–738. 10.1002/ajpa.21118 19890869

[ajp23549-bib-0018] Eadie, E. C. (2015). Ontogeny of foraging competence in capuchin monkeys (*Cebus capucinus*) for easy versus difficult to acquire fruits: A test of the needing to learn hypothesis. PLoS One, 10(9), e0138001. 10.1371/journal.pone.0138001 26372642 PMC4570712

[ajp23549-bib-0019] Fan, P.‐F. , Ai, H.‐S. , Fei, H.‐L. , Zhang, D. , & Yuan, S.‐D. (2013). Seasonal variation of diet and time budget of Eastern hoolock gibbons (*Hoolock leuconedys*) living in a northern montane forest. Primates, 54(2), 137–146. 10.1007/s10329-012-0336-0 23192193

[ajp23549-bib-0020] Fedigan, L. M. , & Jack, K. (2001). Neotropical primates in a regenerating costa rican dry forest: A comparison of howler and capuchin population patterns. International Journal of Primatology, 25(5), 689–713. 10.1023/A:1012092115012

[ajp23549-bib-0021] Fedigan, L. M. , & Jack, K. M. (2012). Tracking neotropical monkeys in Santa Rosa: Lessons from a regenerating Costa Rican Dry Forest. In P. M. Kappeler & D. P. Watts (Eds.), Long‐term field studies of primates (pp. 165–184). Springer. 10.1007/978-3-642-22514-7_8

[ajp23549-bib-0022] Fiore, A. D. (2003). Ranging behavior and foraging ecology of lowland woolly monkeys (*Lagothrix lagotricha poeppigii*) in Yasuní National Park, Ecuador: *Lagothrix* range use and foraging ecology. American Journal of Primatology, 59(2), 47–66. 10.1002/ajp.10065 12619047

[ajp23549-bib-0023] Flores, J. S. , & Ricalde, R. V. (1996). The secretions and exudates of plants used in Mayan traditional medicine. Journal of Herbs, Spices & Medicinal Plants, 4(1), 53–59. 10.1300/J044v04n01_07

[ajp23549-bib-0024] Fragaszy, D. M. , Visalberghi, E. , & Fedigan, L. M. (2004). The complete capuchin: The biology of the genus Cebus. Cambridge University Press.

[ajp23549-bib-0025] De la Fuente, M. F. , Souto, A. , Albuquerque, U. P. , & Schiel, N. (2022). Self‐medication in nonhuman primates: A systematic evaluation of the possible function of the use of medicinal plants, American Journal of Primatology, 84(11), e23438. 10.1002/ajp.23438 36193566

[ajp23549-bib-0026] Gigliarelli, G. , Becerra, J. , Curini, M. , & Marcotullio, M. (2015). Chemical composition and biological activities of Fragrant Mexican Copal (*Bursera* spp.). Molecules, 20(12), Article 12. 10.3390/molecules201219849 PMC6332072 26703535

[ajp23549-bib-0027] Hernandez, A. , MacIntosh, A. , & Huffman, M. (2009). Primate parasite ecology: Patterns and predictions from an ongoing study of Japanese macaques. In M. A. Huffman & C. A. Chapman (Eds.), Primate parasite ecology: The dynamics and study of host‐parasite relationships (pp. 387–401). Cambridge University Press.

[ajp23549-bib-0028] Hogan, J. , & Melin, A. D. (2018). Intra‐ and interannual variation in the fruit diet of wild capuchins: Impact of plant phenology. In U. Kalbitzer & K. M. Jack (Eds.), Primate life histories, sex roles, and adaptability (pp. 193–212). Springer International Publishing. 10.1007/978-3-319-98285-4_10

[ajp23549-bib-0029] Huffman, M. A. (1997). Current evidence for self‐medication in primates: A multidisciplinary perspective. American Journal of Physical Anthropology, 104(S25), 171–200. 10.1002/(SICI)1096-8644(1997)25+<171::AID-AJPA7>3.0.CO;2-7

[ajp23549-bib-0031] Huffman, M. A. (2017). Primate self‐medication, passive prevention and active treatment—A brief review. International Journal of Multidisciplinary Studies, 3(2), Article 2. 10.4038/ijms.v3i2.1

[ajp23549-bib-0032] Huffman, M. A. , Gotoh, S. , Izutsu, D. , Koshimizu, K. , & Kalunde, M. S. (1993). Further observations on the use of the medicinal plant, *Vernonia amygdalina* (Del), by a wild chimpanzee, its possible effect on parasite load, and its phytochemistry. African Study Monographs, 14(4), 227–240.

[ajp23549-bib-0033] Huffman, M. A. , Gotoh, S. , Turner, L. A. , Hamai, M. , & Yoshida, K. (1997). Seasonal trends in intestinal nematode infection and medicinal plant use among chimpanzees in the Mahale Mountains, Tanzania. Primates, 38(2), 111–125. 10.1007/BF02382002

[ajp23549-bib-0034] Janson, C. H. , & Boinski, S. (1992). Morphological and behavioral adaptations for foraging in generalist primates: The case of the cebines. American Journal of Physical Anthropology, 88(4), 483–498. 10.1002/ajpa.1330880405 1503120

[ajp23549-bib-0035] Janzen, D. H. (1988). Management of habitat fragments in a Tropical Dry Forest: Growth. Annals of the Missouri Botanical Garden, 75(1), 105–116. 10.2307/2399468

[ajp23549-bib-0036] Janzen, D. H. (1993). Caterpillar seasonality in a costa rican dry forest. http://copa.acguanacaste.ac.cr:8080/handle/11606/1208

[ajp23549-bib-0037] Janzen, D. H. , & Hallwachs, W. (2020). Área de Conservación Guanacaste, northwestern Costa Rica: Converting a tropical national park to conservation via biodevelopment. Biotropica, 52(6), 1017–1029. 10.1111/btp.12755

[ajp23549-bib-0038] Junor, G. , Porter, R. , Facey, P. , & Yee, T. (2007). Investigation of essential oil extracts from four native jamaican species of Bursera for antibacterial activity. West Indian Medical Journal, 56(1), 22–25. 10.1590/S0043-31442007000100005 17621840

[ajp23549-bib-0039] Kandi, S. , Godishala, V. , Rao, P. , & Ramana, K. V. (2015). Biomedical significance of terpenes: An insight. Biomedicine and Biotechnology, 3(1), Article 1. 10.12691/bb-3-1-2

[ajp23549-bib-0040] Kane, E. E. , Traff, J. N. , Daegling, D. J. , & Scott McGraw, W. (2020). Oral processing behavior of diana monkeys (*Cercopithecus diana*) in Taï National Park, Côte d'Ivoire. Folia Primatologica, 91(6), 541–557. 10.1159/000508072 32492683

[ajp23549-bib-0041] Kano, T. , & Mulavwa, M. (1984). Feeding ecology of the pygmy chimpanzees (*Pan paniscus*) of Wamba. In R. L. Susman (Ed.), The pygmy chimpanzee: Evolutionary biology and behavior (pp. 233–274). Springer. 10.1007/978-1-4757-0082-4_10

[ajp23549-bib-0042] Knott, C. D. (1998). Changes in orangutan caloric intake, energy balance, and ketones in response to fluctuating fruit availability. International Journal of Primatology, 19(6), 1061–1079. 10.1023/A:1020330404983

[ajp23549-bib-0043] Knudsen, J. T. , Eriksson, R. , Gershenzon, J. , & Ståhl, B. (2006). Diversity and distribution of floral scent. The Botanical Review, 72(1), 1–120. 10.1663/0006-8101(2006)72[1:DADOFS]2.0.CO;2

[ajp23549-bib-0044] Koshimizu, K. , Ohigashi, H. , & Huffman, M. A. (1994). Use of *Vernonia amygdalina* by wild chimpanzee: Possible roles of its bitter and related constituents. Physiology & Behavior, 56(6), 1209–1216. 10.1016/0031-9384(94)90368-9 7878093

[ajp23549-bib-0045] Lambert, J. E. (1998). Primate digestion: Interactions among anatomy, physiology, and feeding ecology. Evolutionary Anthropology: Issues, News, and Reviews, 7(1), 8–20. 10.1002/(SICI)1520-6505(1998)7:1<8::AID-EVAN3>3.0.CO;2-C

[ajp23549-bib-0046] Lambert, J. E. (2002). Digestive retention times in forest guenons (*Cercopithecus* spp.) with reference to chimpanzees (*Pan troglodytes*). International Journal of Primatology, 23(6), 1169–1185. 10.1023/A:1021166502098

[ajp23549-bib-0047] Lambert, J. E. , Chapman, C. A. , Wrangham, R. W. , & Conklin‐Brittain, N. L. (2004). Hardness of cercopithecine foods: Implications for the critical function of enamel thickness in exploiting fallback foods. American Journal of Physical Anthropology, 125(4), 363–368. 10.1002/ajpa.10403 15386250

[ajp23549-bib-0048] Lambert, J. E. , & Rothman, J. M. (2015). Fallback foods, optimal diets, and nutritional targets: Primate responses to varying food availability and quality. Annual Review of Anthropology, 44(1), 493–512. 10.1146/annurev-anthro-102313-025928

[ajp23549-bib-0049] Lucas, P. W. , Constantino, P. J. , Chalk, J. , Ziscovici, C. , Wright, B. W. , Fragaszy, D. M. , Hill, D. A. , Lee, J. J. W. , Chai, H. , Darvell, B. W. , Lee, P. K. D. , & Yuen, T. D. B. (2009). Indentation as a technique to assess the mechanical properties of fallback foods. American Journal of Physical Anthropology, 140, 643–652. 10.1002/ajpa.21026 19890850

[ajp23549-bib-0050] Marshall, A. J. , Boyko, C. M. , Feilen, K. L. , Boyko, R. H. , & Leighton, M. (2009). Defining fallback foods and assessing their importance in primate ecology and evolution. American Journal of Physical Anthropology, 140(4), 603–614. 10.1002/ajpa.21082 19890868

[ajp23549-bib-0051] Marshall, A. J. , & Wrangham, R. W. (2007). Evolutionary consequences of fallback foods. International Journal of Primatology, 28(6), 1219–1235. 10.1007/s10764-007-9218-5

[ajp23549-bib-0052] Matthews, J. K. , Ridley, A. , Kaplin, B. A. , & Grueter, C. C. (2020). A comparison of fecal sampling and direct feeding observations for quantifying the diet of a frugivorous primate. Current Zoology, 66(4), 333–343. 10.1093/cz/zoz058 32617082 PMC7319449

[ajp23549-bib-0053] McGraw, W. S. , & Daegling, D. J. (2012). Primate feeding and foraging: Integrating studies of behavior and morphology. Annual Review of Anthropology, 41(1), 203–219. 10.1146/annurev-anthro-092611-145801

[ajp23549-bib-0054] McGraw, W. S. , Vick, A. E. , & Daegling, D. J. (2014). Dietary variation and food hardness in sooty mangabeys (*Cercocebus atys*): Implications for fallback foods and dental adaptation: Seasonality of feeding in Sooty Mangabeys. American Journal of Physical Anthropology, 154(3), 413–423. 10.1002/ajpa.22525 24810136

[ajp23549-bib-0055] Melin, A. D. (2011). Polymorphic colour vision and foraging in white‐faced capuchins: Insights from field research and simulations of monkey vision. PhD thesis. University of Calgary.

[ajp23549-bib-0056] Melin, A. D. , Fedigan, L. M. , Hiramatsu, C. , & Kawamura, S. (2008). Polymorphic color vision in white‐faced capuchins (*Cebus capucinus*): Is there foraging niche divergence among phenotypes. Behavioral Ecology and Sociobiology, 62(5), 659–670. 10.1007/s00265-007-0490-3

[ajp23549-bib-0057] Melin, A. D. , Hiramatsu, C. , Parr, N. A. , Matsushita, Y. , Kawamura, S. , & Fedigan, L. M. (2014). The behavioral ecology of color vision: Considering fruit conspicuity, detection distance and dietary importance. International Journal of Primatology, 35(1), 258–287. 10.1007/s10764-013-9730-8

[ajp23549-bib-0058] Melin, A. D. , Hogan, J. D. , Campos, F. A. , Wikberg, E. , King‐Bailey, G. , Webb, S. , Kalbitzer, U. , Asensio, N. , Murillo‐Chacon, E. , Cheves Hernandez, S. , Guadamuz Chavarria, A. , Schaffner, C. M. , Kawamura, S. , Aureli, F. , Fedigan, L. , & Jack, K. M. (2020). Primate life history, social dynamics, ecology, and conservation: Contributions from long‐term research in Área de Conservación Guanacaste, Costa Rica. Biotropica, 52, 1041–1064. 10.1111/btp.12867

[ajp23549-bib-0059] Melin, A. D. , Webb, S. E. , Williamson, R. E. , & Chiou, K. L. (2018). Data collection in field primatology: A renewed look at measuring foraging behaviour. In U. Kalbitzer & K. M. Jack (Eds.), Primate life histories, sex roles, and adaptability: Essays in honour of Linda M. Fedigan (pp. 161–192). Springer International Publishing. 10.1007/978-3-319-98285-4_9

[ajp23549-bib-0060] Melin, A. D. , Young, H. C. , Mosdossy, K. N. , & Fedigan, L. M. (2014). Seasonality, extractive foraging and the evolution of primate sensorimotor intelligence. Journal of Human Evolution, 71, 77–86. 10.1016/j.jhevol.2014.02.009 24636732

[ajp23549-bib-0061] Mosdossy, K. N. , Melin, A. D. , & Fedigan, L. M. (2015). Quantifying seasonal fallback on invertebrates, pith, and bromeliad leaves by white‐faced capuchin monkeys (*Cebus capucinus*) in a tropical dry forest: Capuchin fallback foods in a Seasonal Dry Forest. American Journal of Physical Anthropology, 158(1), 67–77. 10.1002/ajpa.22767 26010158

[ajp23549-bib-0062] Murthy, K. S. R. , Reddy, M. C. , Rani, S. S. , & Pullaiah, T. (2016). Bioactive principles and biological properties of essential oils of Burseraceae: A review. Journal of Pharmacognosy and Phytochemistry, 5(2), 247–258.

[ajp23549-bib-0063] Nevo, O. , & Heymann, E. W. (2015). Led by the nose: Olfaction in primate feeding ecology. Evolutionary Anthropology: Issues, News, and Reviews, 24(4), 137–148. 10.1002/evan.21458 PMC4584505 26267435

[ajp23549-bib-0064] Nevo, O. , Razafimandimby, D. , Valenta, K. , Jeffrey, J. A. J. , Reisdorff, C. , Chapman, C. A. , Ganzhorn, J. U. , & Ayasse, M. (2019). Signal and reward in wild fleshy fruits: Does fruit scent predict nutrient content. Ecology and Evolution, 9(18), 10534–10543. 10.1002/ece3.5573 31624565 PMC6787828

[ajp23549-bib-0065] Nevo, O. , & Valenta, K. (2018). The ecology and evolution of fruit odor: Implications for primate seed dispersal. International Journal of Primatology, 39(3), 338–355. 10.1007/s10764-018-0021-2

[ajp23549-bib-0066] Ohigashi, H. , Huffman, M. A. , Izutsu, D. , Koshimizu, K. , Kawanaka, M. , Sugiyama, H. , Kirby, G. C. , Warhurst, D. C. , Allen, D. , Wright, C. W. , David Phillipson, J. , Timon‐David, P. , Delmas, F. , Elias, R. , & Balansard, G. (1994). Toward the chemical ecology of medicinal plant use in chimpanzees: The case of *Vernonia amygdalina*, a plant used by wild chimpanzees possibly for parasite‐related diseases. Journal of Chemical Ecology, 20(3), 541–553. 10.1007/BF02059596 24242110

[ajp23549-bib-0067] Oksanen, J. , Simpson, G. , Blanchet, F. , Kindt, R. , Legendre, P. , Minchin, P. , O'Hara, R. , Solymos, P. , Stevens, M. , Szoecs, E. , Wagner, H. , Barbour, M. , Bedward, M. , Bolker, B. , Borcard, D. , Carvalho, G. , Chirico, M. , De Caceres, M. , Durand, S. , & Weedon, J. (2023). Vegan: Community Ecology Package. R package version 2.6‐5. https://github.com/vegandevs/vegan

[ajp23549-bib-0068] Orkin, J. D. , Campos, F. A. , Myers, M. S. , Cheves Hernandez, S. E. , Guadamuz, A. , & Melin, A. D. (2019). Seasonality of the gut microbiota of free‐ranging white‐faced capuchins in a tropical dry forest. The ISME Journal, 13(1), Article 1. 10.1038/s41396-018-0256-0 PMC6298967 30135468

[ajp23549-bib-0069] Owen, K. C. , Melin, A. D. , Campos, F. A. , Fedigan, L. M. , Gillespie, T. W. , & Mennill, D. J. (2020). Bioacoustic analyses reveal that bird communities recover with forest succession in tropical dry forests. Avian Conservation and Ecology, 15(1), art25. 10.5751/ACE-01615-150125

[ajp23549-bib-0070] Parr, N. A. , Fedigan, L. M. , & Kutz, S. J. (2013a). A coprological survey of parasites in white‐faced capuchins (*Cebus capucinus*) from Sector Santa Rosa, ACG, Costa Rica. Folia Primatologica, 84(2), 102–114. 10.1159/000348287 23571310

[ajp23549-bib-0071] Parr, N. A. , Fedigan, L. M. , & Kutz, S. J. (2013b). Predictors of parasitism in wild white‐faced capuchins (*Cebus capucinus* . International Journal of Primatology, 34(6), 1137–1152. 10.1007/s10764-013-9728-2 23571310

[ajp23549-bib-0072] Peraza‐Sánchez, S. R. , & Peña‐Rodríguez, L. M. (1992). Isolation of picropolygamain from the resin of *Bursera simaruba* . Journal of Natural Products, 55(12), 1768–1771. 10.1021/np50090a009 1294696

[ajp23549-bib-0073] Perry, S. (1997). Male‐female social relationships in wild white‐faced capuchins (*Cebus capucinus*). Behaviour, 134(7/8), 477–510.

[ajp23549-bib-0074] Perry, S. , Godoy, I. , & Lammers, W. (2012). The lomas barbudal monkey project: Two decades of research on *Cebus capucinus* . In P. M. Kappeler & D. P. Watts (Eds.), Long‐term field studies of primates (pp. 141–163). Springer. 10.1007/978-3-642-22514-7_7

[ajp23549-bib-0075] Phillips, K. A. (1995). Resource patch size and flexible foraging in white‐faced capuchins (*Cebus capucinus* . International Journal of Primatology, 16(3), 509–519. 10.1007/BF02735800

[ajp23549-bib-0076] Preethi, S. , Jain, V. , Patil, A. B. , Siree, K. G. , & Sowmya, A. (2020). Restorative efficiency of *Bursera simaruba*‐isolated phytonutrients for the therapy of various diseases. Systematic Reviews in Pharmacy: Journal of Pharmacy Education and Practice, 11(11), 1022–1036.

[ajp23549-bib-0077] R Core Team, R. (2022). R: A language and environment for statistical computing. R Foundation for Statistical Computing.

[ajp23549-bib-0078] Reed, D. A. , & Ross, C. F. (2010). The influence of food material properties on jaw kinematics in the primate, *Cebus* . Archives of Oral Biology, 55(12), 946–962. 10.1016/j.archoralbio.2010.08.008 20880517

[ajp23549-bib-0079] Rodriguez, E. , & Wrangham, R. (1993). Zoopharmacognosy: The use of medicinal plants by animals. In K. R. Downum , J. T. Romeo & H. A. Stafford (Eds.), Phytochemical potential of tropical plants (pp. 89–105). Springer. 10.1007/978-1-4899-1783-6_4

[ajp23549-bib-0080] Romero‐Estrada, A. , Maldonado‐Magaña, A. , González‐Christen, J. , Bahena, S. M. , Garduño‐Ramírez, M. L. , Rodríguez‐López, V. , & Alvarez, L. (2016). Anti‐inflammatory and antioxidative effects of six pentacyclic triterpenes isolated from the Mexican copal resin of Bursera copallifera. BMC Complementary and Alternative Medicine, 16(1), 422. 10.1186/s12906-016-1397-1 27784308 PMC5081879

[ajp23549-bib-0081] Rosenberger, A. L. (1992). Evolution of feeding niches in new world monkeys. American Journal of Physical Anthropology, 88(4), 525–562. 10.1002/ajpa.1330880408 1503123

[ajp23549-bib-0082] Rosenberger, A. L. (2013). Fallback foods, preferred foods, adaptive zones, and primate origins. American Journal of Primatology, 75(9), 883–890. 10.1002/ajp.22162 23630044

[ajp23549-bib-0083] Rothman, J. M. , Chapman, C. A. , & Van Soest, P. J. (2012). Methods in primate nutritional ecology: A user's guide. International Journal of Primatology, 33(3), 542–566. 10.1007/s10764-011-9568-x

[ajp23549-bib-0084] Rothman, J. M. , Dierenfeld, E. S. , Molina, D. O. , Shaw, A. V. , Hintz, H. F. , & Pell, A. N. (2006). Nutritional chemistry of foods eaten by gorillas in Bwindi Impenetrable National Park, Uganda. American Journal of Primatology, 68(7), 675–691. 10.1002/ajp.20243 16550527

[ajp23549-bib-0085] Schmitt, M. H. , Shuttleworth, A. , Ward, D. , & Shrader, A. M. (2018). African elephants use plant odours to make foraging decisions across multiple spatial scales. Animal Behaviour, 141, 17–27. 10.1016/j.anbehav.2018.04.016

[ajp23549-bib-0086] Serckx, A. , Kühl, H. S. , Beudels‐Jamar, R. C. , Poncin, P. , Bastin, J.‐F. , & Huynen, M.‐C. (2015). Feeding ecology of bonobos living in forest‐savannah mosaics: Diet seasonal variation and importance of fallback foods. American Journal of Primatology, 77(9), 948–962. 10.1002/ajp.22425 25974229 PMC7159761

[ajp23549-bib-0087] Shen, V. K. , Siderius, D. W. , Krekelberg, W. P. , & Hatch, H. W. (2014). NIST standard reference simulation website. NIST standard reference database number, 173. https://www.nist.gov/programs-projects/nist-standard-reference-simulation-website

[ajp23549-bib-0088] Singer, M. S. , & Bernays, E. A. (2003). Understanding omnivory needs a behavioral perspective. Ecology, 84(10), 2532–2537. 10.1890/02-0397

[ajp23549-bib-0089] Sosa, S. , Balick, M. J. , Arvigo, R. , Esposito, R. G. , Pizza, C. , Altinier, G. , & Tubaro, A. (2002). Screening of the topical anti‐inflammatory activity of some Central American plants. Journal of Ethnopharmacology, 81(2), 211–215. 10.1016/S0378-8741(02)00080-6 12065153

[ajp23549-bib-0090] Springer, A. , & Kappeler, P. M. (2016). Intestinal parasite communities of six sympatric lemur species at Kirindy Forest, Madagascar. Primate Biology, 3(2), 51–63. 10.5194/pb-3-51-2016

[ajp23549-bib-0091] Stevenson, P. R. , Quinones, M. J. , & Ahumada, J. A. (2000). Influence of fruit availability on ecological overlap among four neotropical primates at Tinigua National Park, Colombia1. Biotropica, 32(3), 533–544. 10.1111/j.1744-7429.2000.tb00499.x

[ajp23549-bib-0092] Terborgh, J. (1983). Five new world primates: A study in comparative ecology. Princeton University Press.

[ajp23549-bib-0093] Theis, N. , & Lerdau, M. (2003). The evolution of function in plant secondary metabolites. International Journal of Plant Sciences, 164(S3), S93–S102. 10.1086/374190

[ajp23549-bib-0094] Thiery, G. , Guy, F. , & Lazzari, V. (2017). Investigating the dental toolkit of primates based on food mechanical properties: Feeding action does matter. American Journal of Primatology, 79(6), e22640. 10.1002/ajp.22640 28150439

[ajp23549-bib-0095] Tutin, C. E. G. , Ham, R. M. , White, L. J. T. , & Harrison, M. J. S. (1997). The primate community of the Lopé reserve, Gabon: Diets, responses to fruit scarcity, and effects on biomass. American Journal of Primatology, 42(1), 1–24. 9108968 10.1002/(SICI)1098-2345(1997)42:1<1::AID-AJP1>3.0.CO;2-0

[ajp23549-bib-0096] Venables, W. N. , & Ripley, B. D. (2002). Modern applied statistics with S (4th ed.). Springer. https://www.stats.ox.ac.uk/pub/MASS4/

[ajp23549-bib-0097] Weiß, B. M. , Marcillo, A. , Manser, M. , Holland, R. , Birkemeyer, C. , & Widdig, A. (2018). A non‐invasive method for sampling the body odour of mammals. Methods in Ecology and Evolution, 9(2), 420–429. 10.1111/2041-210X.12888

[ajp23549-bib-0098] Wood, S. N. (2011). Fast stable restricted maximum likelihood and marginal likelihood estimation of semiparametric generalized linear models. Journal of the Royal Statistical Society Series B: Statistical Methodology, 73(1), 3–36.

[ajp23549-bib-0099] Wrangham, R. W. , Conklin‐Brittain, N. L. , & Hunt, K. D. (1998). Dietary response of chimpanzees and cercopithecines to seasonal variation in fruit abundance. I. Antifeedants. International Journal of Primatology, 19(6), 949–970. 10.1023/A:1020318102257

[ajp23549-bib-0100] Wrangham, R. W. , Conklin, N. L. , Chapman, C. A. , Hunt, K. D. , Milton, K. , Rogers, E. , Whiten, A. , & Barton, R. A. (1991). The significance of fibrous foods for kibale forest chimpanzees [and discussion]. Philosophical Transactions: Biological Sciences, 334(1270), 171–178. 1685575 10.1098/rstb.1991.0106

[ajp23549-bib-0101] Wright, B. (2005). Craniodental biomechanics and dietary toughness in the genus. Journal of Human Evolution, 48(5), 473–492. 10.1016/j.jhevol.2005.01.006 15857651

[ajp23549-bib-0102] Wright, B. W. , Wright, K. A. , Chalk, J. , Verderane, M. P. , Fragaszy, D. , Visalberghi, E. , Izar, P. , Ottoni, E. B. , Constantino, P. , & Vinyard, C. (2009). Fallback foraging as a way of life: Using dietary toughness to compare the fallback signal among capuchins and implications for interpreting morphological variation. American Journal of Physical Anthropology, 140(4), 687–699. 10.1002/ajpa.21116 19890863

[ajp23549-bib-0103] Yamashita, N. , Vinyard, C. J. , & Tan, C. L. (2009). Food mechanical properties in three sympatric species of Hapalemur in Ranomafana National Park, Madagascar. American Journal of Physical Anthropology, 139(3), 368–381. 10.1002/ajpa.20992 19115398

