# Supplementary figures and images for: Picking pithy plants: Pith selectivity by wild white‐faced capuchin monkeys, Cebus imitator
Source: Am J Primatol. 2023 Sep 10;87(1):e23549. doi: 10.1002/ajp.23549 (PMC11650937; doi:10.1002/ajp.23549)

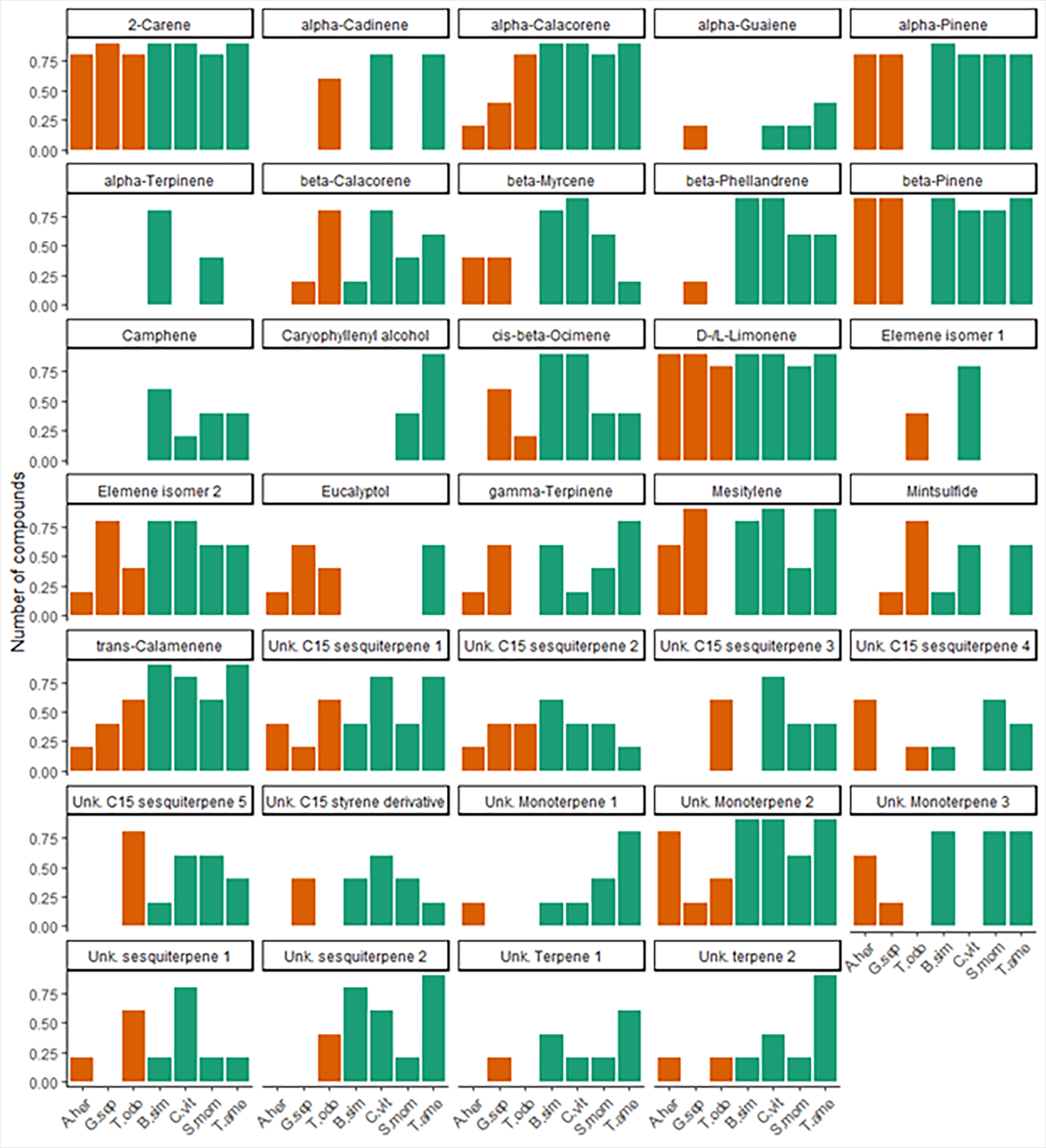

Supplement: Supplementary file 2 — Suppl. Fig. S1. Proportion of terpenoid VOCs in the pith samples. The sample size for each species was n = 5, except for S. mombin and T. odontadeniiflora (n = 4). [file AJP-87-e23549-s002.tif]
