# Supplementary material for: Picking pithy plants: Pith selectivity by wild white‐faced capuchin monkeys, Cebus imitator
Source: Am J Primatol. 2023 Sep 10;87(1):e23549. doi: 10.1002/ajp.23549 (PMC11650937; doi:10.1002/ajp.23549)
Supplement: Supplementary file 3 — Supporting information. [file AJP-87-e23549-s003.docx]

**Suppl. Table S1.** List of dietary and non-dietary species used in this study, for each of the following pith properties measured: relative pith quantity, mechanical hardness, odor profile, and macronutritional profile.

| **Species** | **Dietary/ Non-dietary** | **Quantity** | **Hardness** | **Odor** | **Nutrient** |
| --- | --- | --- | --- | --- | --- |
| *Ateleia herbert-smithii* | Non-dietary | ✔ | ✔ | ✔ | ✔ |
| *Bursera simaruba* | Dietary | ✔ | ✔ | ✔ | ✔ |
| *Cochlospermum vitifolium* | Dietary | ✔ | ✔ | ✔ | ✔ |
| *Gliricidia sepium* | Non-dietary | ✔ | ✔ | ✔ | ✔ |
| *Lonchocarpus miniflorus* | Non-dietary | ✔ | ✔ | O | O |
| *Luehea speciosa* | Non-dietary | ✔ | ✔ | O | O |
| *Mabea occidentalis* | Dietary | ✔ | ✔ | O | O |
| *Rehdera trinervis* | Non-dietary | ✔ | ✔ | O | O |
| *Spondias mombin* | Dietary | ✔ | ✔ | ✔ | ✔ |
| *Tabebuia ochracea* | Non-dietary | ✔ | ✔ | O | O |
| *Tabernaemontana odontadeniiflora* | Non-dietary | ✔ | ✔ | ✔ | ✔ |
| *Trichilia americana* | Dietary | ✔ | ✔ | ✔ | ✔ |

**Suppl. Table S2.** Description of pith odor sampling, gas chromatography-mass spectrometry (GC-MS) analyses, GC-MS data interpretation and VOC identification, used in this study.

| **Step** | **Description** |
| --- | --- |
| Odor sampling | - Volatile organic compounds (VOCs) extracted from pith samples using a semi-static headspace procedure. - Sampling material: chromatoprobe VOC traps made of a quartz tube (20 mm x 3 mm) containing three adsorbent media (1.5 mg Tenax TA, 1.5 mg Carbotrap and 1.5 mg Carbosieve S-III, Sigma-Aldrich) trapped between layers of glass wool. These absorbents, in particular Tenax and Carbosieve S-III, have a high and stable affinity for VOCs, and high shelf stability at room temperature. - Prior to use, probes sterilized in a bath of methanol for 24 h, followed by 2 h baking at 100ºC, and stored in a clean glass jar (Supelco). - Odor sampling procedure:  1. Incubate each pith sample into a 25 x 40 cm sealed sampling bag (Toppits) at room temperature (ca. 30ºC) for 60 minutes. 2. Insert the probe into the bag opening, mounted on a Teflon tube connected to a membrane pump 3. Extract the headspace inside the bag for 30 minutes at a flow rate of 0.2 L/min. 4. Store probe into individual 2 mL glass vials fitted with an insert and screw caps. 5. Keep cold until transportation.  - For comparison, control samples additionally collected in the same conditions: empty bag, nitrile glove, paper towel and empty probes. - Probe samples stored on-site at 4ºC for one week, then shipped at room temperature to Ulm University, Germany, for GC-MS analyses. |
| GC-MS analyses | - Probes analyzed using thermal desorption (thermal desorption unit, TDU) and an Agilent GC 7890B equipped with a DB-WAX polar capillary column (30 m × 0.25 mm × 0.25 µm, Agilent) and a cold injection system (CSI4, Gerstel 6817-U glass liner filled with silanized glass wool), coupled with an Agilent MS 5977A operated on electron ionization (scan range 35-450 Da). - Helium used as carrier gas at a flow rate of 1 mL/min. - Procedure:  1. Introduce sample to the TDU at 30°C in splitless mode, held for 1 min. 2. Heat up TDU at 100°C/min to 310°C, held for 8 min. 3. Liner cooled to −100°C using liquid nitrogen. 4. After transfer to the liner, heat it up at 12°C/min to 250°C, held for 8 min. 5. Initial oven temperature set to 30°C, held for 1 min and then increased by 10°C/min to 240°C, held for 30 min. 6. MS transfer line temperature set to 250°C, MS source temperature to 230°C and MS quadrupole temperature to 150°C. |
| GC-MS data interpretation | - GC-MS data processed using a semi-automatic procedure:  1. Automatic peak detection, deconvolution and integration using AMDIS 2.73. Deconvolution parameters used: medium resolution, low sensitivity and high peak shape requirement. 2. Peaks with similar retention times (RTs) grouped into RT ranges using custom-made R-script. 3. Manual correction of peak RT ranges using information of mass-to-charge ratios. 4. Targeted reverse library search in AMDIS to search for selected peaks in all the chemical profiles (because some low abundance compounds may not have been detected in the first AMDIS search). 5. Manual exclusion from further analyses:   5.1. Peaks strongly identified as contaminants,  5.2. Rare peaks (i.e., present in fewer than 4 samples),  5.3. Peaks found in higher amounts in at least one blank sample, and peaks with average area < 0.2% compound of highest area (to avoid the inclusion of background noise).   - In our dataset of 33 samples: 289 peaks initially detected; 122 peaks retained for statistical analyses and compound identification. |
| VOC identification | - VOC identification based on retention times and mass spectra using NIST14 - Use of additional information of retention indices, calculated based on a series of C9-C30 n-alkane reference mixture analyzed under identical conditions. - When exact compound identification not possible in NIST, attempt made to identify its main chemical characteristics. - Compounds classified into six broad chemical classes: aliphatics, benzenoids and phenylpropanoids, terpenoids, nitrogen-containing compounds, miscellaneous cyclic compounds, and unidentified. |

**Suppl. Table S3**. List of the 122 volatile organic compounds (VOCs) found in pith samples analyzed by thermal desorption–gas chromatography-mass spectrometry. RT = retention time in min.

| **VOC #** | **Mean RT** | **Std Dev RT** | **Tentative Identity** | **Chemical Class** |
| --- | --- | --- | --- | --- |
| 1 | 1.94 | 0.01 | 3-Methylpentane | Aliphatics |
| 2 | 2.39 | 0.04 | Acetaldehyde | Aliphatics |
| 3 | 2.82 | 0.13 | Acetone | Aliphatics |
| 4 | 3.84 | 0.04 | Ethanol | Aliphatics |
| 5 | 4.18 | 0.05 | Branched C10 alkane | Aliphatics |
| 6 | 4.41 | 0.02 | Dimethyl carbonate | Aliphatics |
| 7 | 4.79 | 0.02 | Acetonitrile | Nitrogen-containing compounds |
| 8 | 4.90 | 0.04 | alpha-Pinene | Terpenoids |
| 9 | 5.10 | 0.03 | Toluene | Benzenoids and phenylpropanoids |
| 10 | 5.46 | 0.01 | Camphene | Terpenoids |
| 11 | 5.66 | 0.01 | Hexanal | Aliphatics |
| 12 | 5.71 | 0.03 | Undecane | Aliphatics |
| 13 | 5.99 | 0.07 | beta-Pinene | Terpenoids |
| 14 | 6.22 | 0.05 | Ethylbenzene | Benzenoids and phenylpropanoids |
| 15 | 6.42 | 0.01 | 2-Carene | Terpenoids |
| 16 | 6.58 | 0.02 | beta-Myrcene | Terpenoids |
| 17 | 6.75 | 0.01 | (1-Methylethyl)-benzene | Benzenoids and phenylpropanoids |
| 18 | 6.75 | 0.01 | Unknown monoterpene 1 | Terpenoids |
| 19 | 6.78 | 0.02 | alpha-Terpinene | Terpenoids |
| 20 | 6.90 | 0.01 | Branched C12 alkane | Aliphatics |
| 21 | 7.00 | 0.01 | D-/L-Limonene | Terpenoids |
| 22 | 7.11 | 0.01 | beta-Phellandrene | Terpenoids |
| 23 | 7.17 | 0.02 | Propylbenzene | Benzenoids and phenylpropanoids |
| 24 | 7.35 | 0.01 | Unknown branched benzene | Benzenoids and phenylpropanoids |
| 25 | 7.37 | 0.01 | Eucalyptol | Terpenoids |
| 26 | 7.38 | 0.01 | Unknown compound 1 | Unidentified |
| 27 | 7.53 | 0.02 | cis-beta-Ocimene | Terpenoids |
| 28 | 7.77 | 0.01 | gamma-Terpinene | Terpenoids |
| 29 | 7.80 | 0.03 | Styrene | Benzenoids and phenylpropanoids |
| 30 | 7.81 | 0.01 | 3-Octanone | Aliphatics |
| 31 | 7.84 | 0.02 | Unknown monoterpene 2 | Terpenoids |
| 32 | 7.90 | 0.02 | Branched C13 alkane | Aliphatics |
| 33 | 8.00 | 0.04 | Mesitylene | Terpenoids |
| 34 | 8.27 | 0.07 | Octanal | Aliphatics |
| 35 | 8.23 | 0.06 | Unknown monoterpene 3 | Terpenoids |
| 36 | 8.48 | 0.07 | Unknown compound 2 | Unidentified |
| 37 | 8.46 | 0.06 | Unknown alkene | Aliphatics |
| 38 | 8.99 | 0.03 | Branched C14 alkane | Aliphatics |
| 39 | 9.13 | 0.06 | 6-Methyl-5-hepten-2-one | Aliphatics |
| 40 | 9.22 | 0.10 | 1-Ethyl-2,4-dimethylbenzene | Benzenoids and phenylpropanoids |
| 41 | 9.33 | 0.14 | Branched C9 aldehyde | Aliphatics |
| 42 | 9.55 | 0.16 | 1-Hydroxy-2-propanone | Aliphatics |
| 43 | 9.61 | 0.03 | 1-Hexanol | Aliphatics |
| 44 | 10.00 | 0.02 | Unknown terpene 1 | Terpenoids |
| 45 | 10.16 | 0.02 | Unknown compound 3 | Unidentified |
| 46 | 10.34 | 0.09 | Unknown C15 sesquiterpene 1 | Terpenoids |
| 47 | 10.25 | 0.04 | Unknown C15 styrene derivative | Terpenoids |
| 48 | 10.41 | 0.02 | Elemene isomer 1 | Terpenoids |
| 49 | 10.49 | 0.03 | Elemene isomer 2 | Terpenoids |
| 50 | 10.62 | 0.05 | Unknown C15 sesquiterpene 2 | Terpenoids |
| 51 | 10.81 | 0.07 | 1-Octen-3-ol | Aliphatics |
| 52 | 11.41 | 0.07 | Unknown C15 sesquiterpene 3 | Terpenoids |
| 53 | 11.52 | 0.02 | Unknown C15 sesquiterpene 4 | Terpenoids |
| 54 | 11.59 | 0.04 | Pentadecane | Aliphatics |
| 55 | 11.77 | 0.04 | Branched C16 alkane 1 | Aliphatics |
| 56 | 11.74 | 0.01 | 2-Nonenal | Aliphatics |
| 57 | 12.06 | 0.12 | Branched C16 alkane 2 | Aliphatics |
| 58 | 12.04 | 0.06 | Propanoic acid | Aliphatics |
| 59 | 12.65 | 0.03 | alpha-Guaiene | Terpenoids |
| 60 | 12.88 | 0.02 | Unknown compound 4 | Unidentified |
| 61 | 13.10 | 0.03 | Butanoic acid | Aliphatics |
| 62 | 13.56 | 0.03 | 2-Methylbutanoic acid | Aliphatics |
| 63 | 14.01 | 0.02 | Unknown compound 5 | Unidentified |
| 64 | 14.43 | 0.01 | Unknown N compound 1 | Nitrogen-containing compounds |
| 65 | 14.55 | 0.05 | 2(5H)-Furanone | Miscellaneous cyclic compounds |
| 66 | 14.66 | 0.05 | Unknown sesquiterpene 1 | Terpenoids |
| 67 | 14.66 | 0.02 | 2-Hydroxy-2-cyclopenten-1-one | Miscellaneous cyclic compounds |
| 68 | 14.84 | 0.02 | Unknown N compound 2 | Nitrogen-containing compounds |
| 69 | 14.86 | 0.02 | Unknown N compound 3 | Nitrogen-containing compounds |
| 70 | 14.93 | 0.02 | Unknown N compound 4 | Nitrogen-containing compounds |
| 71 | 15.07 | 0.04 | alpha-Cadinene | Terpenoids |
| 72 | 15.12 | 0.03 | Tridecan-2-one | Aliphatics |
| 73 | 15.12 | 0.02 | Unknown cyclic alcohol | Aliphatics |
| 74 | 15.23 | 0.05 | trans-Calamenene | Terpenoids |
| 75 | 15.42 | 0.02 | Hexanoic acid | Aliphatics |
| 76 | 15.63 | 0.02 | 2-Methoxyphenol (= Guaiacol) | Benzenoids and phenylpropanoids |
| 77 | 15.77 | 0.01 | Unknown N compound 5 | Nitrogen-containing compounds |
| 78 | 16.21 | 0.13 | alpha-Calacorene | Terpenoids |
| 79 | 16.11 | 0.08 | Butylated hydroxytoluene | Benzenoids and phenylpropanoids |
| 80 | 16.61 | 0.01 | 2-Ethylhexanoic acid | Aliphatics |
| 81 | 16.70 | 0.02 | Unknown C15 sesquiterpene 5 | Terpenoids |
| 82 | 16.81 | 0.01 | Maltol | Miscellaneous cyclic compounds |
| 83 | 16.82 | 0.02 | beta-Calacorene | Terpenoids |
| 84 | 17.01 | 0.01 | Levoglucosenone | Miscellaneous cyclic compounds |
| 85 | 17.20 | 0.07 | Caryophyllene oxide | Miscellaneous cyclic compounds |
| 86 | 17.20 | 0.01 | Furyl hydroxymethyl ketone | Miscellaneous cyclic compounds |
| 87 | 17.41 | 0.01 | Salvial-4(14)-en-1-one | Miscellaneous cyclic compounds |
| 88 | 17.47 | 0.01 | Unknown compound 6 | Unidentified |
| 89 | 17.56 | 0.02 | 1,2,4-Trimethoxybenzene | Benzenoids and phenylpropanoids |
| 90 | 17.69 | 0.04 | (1-Methylundecyl)-benzene | Benzenoids and phenylpropanoids |
| 91 | 17.79 | 0.01 | Caryophyllenyl alcohol | Terpenoids |
| 92 | 17.81 | 0.01 | Tridecan-1-ol | Aliphatics |
| 93 | 18.04 | 0.02 | Unknown alcohol | Aliphatics |
| 94 | 18.13 | 0.02 | Heneicosane | Aliphatics |
| 95 | 18.22 | 0.01 | 4,5-Dimethyl-1,3-dioxol-2-one | Miscellaneous cyclic compounds |
| 96 | 18.42 | 0.03 | Unknown sesquiterpene 2 | Terpenoids |
| 97 | 18.73 | 0.01 | 2-Hydroxy-gamma-butyrolactone | Miscellaneous cyclic compounds |
| 98 | 18.84 | 0.01 | 3-Pyridinol | Nitrogen-containing compounds |
| 99 | 18.87 | 0.01 | Mintsulfide | Terpenoids |
| 100 | 18.98 | 0.01 | 2-Methoxy-4-vinylphenol | Benzenoids and phenylpropanoids |
| 101 | 19.27 | 0.01 | 4-Methyl-1H-imidazole | Nitrogen-containing compounds |
| 102 | 19.35 | 0.01 | 2-Heptadecanone | Aliphatics |
| 103 | 19.60 | 0.01 | 2,6-Dimethoxyphenol | Benzenoids and phenylpropanoids |
| 104 | 20.03 | 0.01 | Unknown terpene 2 | Terpenoids |
| 105 | 20.19 | 0.01 | Glutarimide | Nitrogen-containing compounds |
| 106 | 20.36 | 0.01 | trans-Isoeugenol | Benzenoids and phenylpropanoids |
| 107 | 20.65 | 0.01 | 1-Hexadecanol | Aliphatics |
| 108 | 20.82 | 0.01 | 1,4:3,6-Dianhydro-alpha-d-glucopyranose | Miscellaneous cyclic compounds |
| 109 | 21.31 | 0.01 | Branched C25 alkane | Aliphatics |
| 110 | 21.51 | 0.01 | Unknown compound 7 | Unidentified |
| 111 | 22.14 | 0.01 | Branched C26 alkane | Aliphatics |
| 112 | 22.25 | 0.06 | Dihydromyristicin | Benzenoids and phenylpropanoids |
| 113 | 22.23 | 0.01 | 2-(3,4-Dimethoxyphenyl)-3,4-dihydro-6-methyl-2H-1-benzopyran-3,4-diol | Benzenoids and phenylpropanoids |
| 114 | 22.32 | 0.02 | Unknown compound 8 | Unidentified |
| 115 | 22.39 | 0.02 | 1-Octadecanol | Aliphatics |
| 116 | 22.46 | 0.04 | Unknown methoxyphenyl acetone | Benzenoids and phenylpropanoids |
| 117 | 22.73 | 0.06 | Unknown compound 9 | Unidentified |
| 118 | 23.65 | 0.01 | 5-Acetamino-2-methylphenyl acetate | Nitrogen-containing compounds |
| 119 | 23.72 | 0.02 | Unknown compound 10 | Unidentified |
| 120 | 24.33 | 0.01 | Unknown N compound 6 | Nitrogen-containing compounds |
| 121 | 25.70 | 0.16 | Branched C29 alkane | Aliphatics |
| 122 | 32.84 | 0.87 | Tetratriacontane | Aliphatics |

**Suppl. Table S4**. Number of pith patch visits recorded per taxon from June 2018-February 2022.

| **Tree species** | **Number of pith patch visits** |
| --- | --- |
| *Bursera simaruba* | 241 |
| *Mabea occidentalis* | 19 |
| *Vachellia collinsi* | 19 |
| *Trichilia americana* | 11 |
| Unidentified species | 7 |
| *Cochlospermum vitifolium* | 5 |
| *Trichilia martiana* | 5 |
| *Spondias mombin* | 1 |
| *Astronium graveolens* | 1 |
| *Maclura tinctoria* | 1 |
| *Serjania schiedeana* | 1 |
| *Xylosma flexuosa* | 1 |


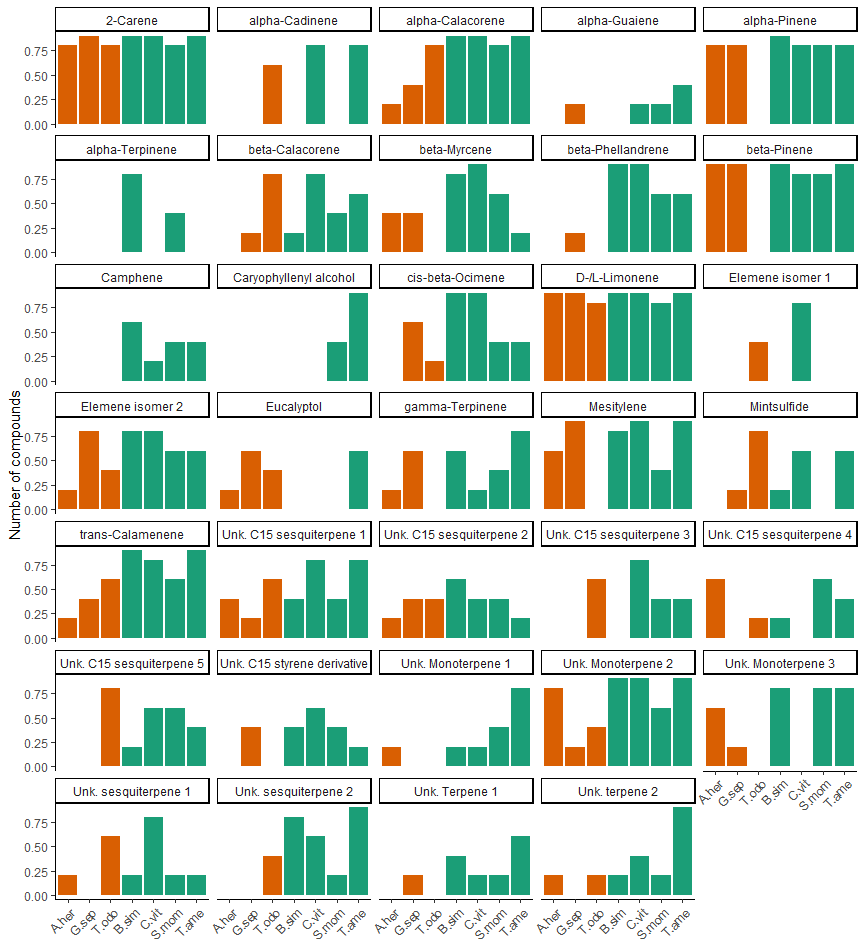


**Suppl. Fig. S1.** Proportion of terpenoid VOCs in the pith samples. The sample size for each species was n = 5, except for *S. mombin* and *T. odontadeniiflora* (n = 4).
